# CD44 Is a Negative Cell Surface Marker for Pluripotent Stem Cell Identification during Human Fibroblast Reprogramming

**DOI:** 10.1371/journal.pone.0085419

**Published:** 2014-01-09

**Authors:** Rene H. Quintanilla, Joanna S. T. Asprer, Candida Vaz, Vivek Tanavde, Uma Lakshmipathy

**Affiliations:** 1 Cell Biology & Stem Cell Sciences, Life Technologies, Carlsbad, California, United States of America; 2 Bioinformatics Institute, Agency for Science Technology & Research (A*Star), Singapore, Republic of Singapore; 3 Institute for Medical Biology, Agency for Science Technology & Research (A*Star), Singapore, Republic of Singapore; Stem Cell Research Institute, Belgium

## Abstract

Induced pluripotent stem cells (iPSCs) are promising tools for disease research and cell therapy. One of the critical steps in establishing iPSC lines is the early identification of fully reprogrammed colonies among unreprogrammed fibroblasts and partially reprogrammed intermediates. Currently, colony morphology and pluripotent stem cell surface markers are used to identify iPSC colonies. Through additional clonal characterization, we show that these tools fail to distinguish partially reprogrammed intermediates from fully reprogrammed iPSCs. Thus, they can lead to the selection of suboptimal clones for expansion. A subsequent global transcriptome analysis revealed that the cell adhesion protein CD44 is a marker that differentiates between partially and fully reprogrammed cells. Immunohistochemistry and flow cytometry confirmed that CD44 is highly expressed in the human parental fibroblasts used for the reprogramming experiments. It is gradually lost throughout the reprogramming process and is absent in fully established iPSCs. When used in conjunction with pluripotent cell markers, CD44 staining results in the clear identification of fully reprogrammed cells. This combination of positive and negative surface markers allows for easier and more accurate iPSC detection and selection, thus reducing the effort spent on suboptimal iPSC clones.

## Introduction

Reprogramming technologies enable a variety of somatic cells, including fibroblasts and lymphocytes, to enter an embryonic stem cell (ESC)-like state, resulting in the generation of induced pluripotent stem cells (iPSC) [1], [2], [3], [4]. Studies so far indicate that reprogramming is a complex process with intermediate stages of reprogramming characterized by unique gene expression patterns [5], [6], [7], [8], [9], [10], [11]. For fibroblast reprogramming, these intermediates were recently reported to transiently upregulate epidermal genes, while downregulating fibroblast genes and upregulating pluripotency genes [12].

The reprogramming process takes weeks and necessitates the early identification and isolation of fully reprogrammed iPSC colonies from a master plate that is often dominated by unreprogrammed fibroblasts and partially reprogrammed intermediates [13]. Current methods for isolation include visual inspection of colony morphology or staining for stem cell surface markers like Stage Specific Embryonic Antigen (SSEA4) and the Tumor Rejection Antigens (TRA-1-60, TRA-1-81) [14], [15], [16]. Alkaline phosphatase (AP) staining is also used for pluripotent stem cell (PSC) identification although common protocols compromise cell integrity and are therefore only applicable for terminal staining [17], [18], [19]. Recently, we reported a novel method for staining AP positive cells while preserving cell health [20].

The above methods can distinguish between unreprogrammed parental somatic cells and reprogrammed cells. However, they fail to distinguish fully reprogrammed iPSCs from partially reprogrammed intermediates that often form similar colonies and express pluripotency genes [13], [21]. Double staining with positive and negative PSC markers offers a more robust method for distinguishing fully reprogrammed iPSCs from reprogramming intermediates. Such a combination of markers has previously been utilized to refine the identification of fully reprogrammed colonies based on SSEA4, TRA-1-60, and the fibroblast marker CD13 [21].

Here, we generated and characterized 8 fully reprogrammed and 2 partially reprogrammed samples. We then screened for differentially expressed markers in a global gene expression study comparing these samples as well as parental fibroblasts and ESCs. One marker that was differentially expressed between pluripotent cells and partially reprogrammed or unreprogrammed fibroblasts was CD44. CD44 is a surface glycoprotein that serves as a receptor for hyaluronic acid [22]. It is expressed in a wide variety of cell types, including epithelial cells and lymphocytes [23], [24], and is involved in cell adhesion, cell migration, lymphocyte homing and epithelial-mesenchymal transitions [23], [25].

Utilizing indirect immunofluorescence and flow cytometry, we confirmed that CD44 is robustly expressed in human fibroblasts, is gradually lost as reprogramming progresses, and is absent in established PSCs. While clones may form distinct colonies and express pluripotency markers, they vary in their degree of reprogramming and expression of CD44. The combined use of the negative selection marker CD44 and the positive pluripotency marker SSEA4 allows easier and earlier detection of fully reprogrammed iPSC colonies.

## Materials and Methods

All reagents were from Life Technologies unless otherwise stated.

### Cells

Human H9 Embryonic Stem Cells (WA09) obtained from WiCell Research Institute and the Gibco® Episomal human iPSC line are commercially available. Human neonatal foreskin fibroblasts (BJ strain) were purchased from ATCC® and were grown in fibroblast media comprised of DMEM, 10% ESC-Qualified FBS, and 10 mM MEM Non Essential Amino Acids (MEM-NEAA). Mitotically inactivated murine embryonic fibroblasts (MEFs) were purchased from Millipore and grown on Attachment Factor Protein (Collagen) in DMEM media containing 10% ESC cell qualified FBS, 10 mM MEM-NEAA solution and 55 mM 2-Mercaptoethanol.

### Human ESC and iPSC culture

Feeder-dependent H9 ESCs, commercially available iPSC lines, and internally generated iPSCs were cultured in human iPSC media comprising of DMEM/F-12 media containing 20% KnockOut^TM^ Serum Replacement (KSR), 10 mM MEM-NEAA solution, 55 mM 2-Mercaptoethanol and 4 ng/ml basic FGF on MEF feeder layers and maintained in a 5% CO_2_, 37°C, humidified incubator. For feeder-free cultures, ESCs and iPSCs were cultured in StemPro® hESC SFM supplemented with 100 mM 2-Mercaptoethanol and 8 ng/ml basic FGF, and grown on LDEV-free hESC qualified reduced growth factor basement membrane matrix Geltrex®, coated onto tissue culture treated surfaces. ESCs and iPSCs were routinely passaged using Collagenase, Type IV. iPSC clones were mechanically picked and propagated during the early stages of reprogramming.

### Reprogramming

BJ fibroblasts were seeded at appropriate plating densities onto tissue culture-treated dishes in fibroblast media. The cells were transduced overnight with the CytoTune®-iPS Sendai reprogramming kit using a multiplicity of infection (MOI) of 3 for each factor-containing virus (OKSM), according to the product manual. Overnight transductions performed on BJ fibroblasts using CytoTune®-iPS 2.0 Sendai reprogramming kit used a MOI of 5-5-3 for the viruses encoding KOS, hc-myc, and hKlf4, respectively. Seven days post-transduction, cells were harvested in single cell suspension and reseeded at the desired densities onto MEF feeders in human iPSC media or onto Geltrex™ and StemPro® hESC SFM. Compact colonies with distinct features of pluripotent stem cells start to emerge at around 2 weeks post- transduction. Colonies at 21 to 28 days post-transduction were identified based on robust alkaline phosphatase activity using AP Live Stain. Individual clones were scored using a 27-gauge needle, picked manually and expanded further.

Epi5^TM^ Episomal iPSC reprogramming and mRNA-based (StemGent) reprogramming was carried out according to the manufacturers' instructions. For more details, refer to Methods S1.

Reprogramming experiments were performed using the CytoTune®-iPS Sendai reprogramming kit unless otherwise stated.

### Karyotyping Analysis

Karyotyping services were provided by Cell Line Genetics®. Results were based on cytogenetic analysis performed using GTL-banding technique on twenty G-banded metaphase cells.

### AP Staining

ESC and iPSC cultures were washed twice with DMEM/F-12 media prior to live AP staining. Cultures were then incubated for 20 minutes with AP Live Stain diluted 1∶500 in DMEM/F-12. Following incubation, the cultures were washed three times with DMEM/F-12 and visualized for green fluorescent-labeled colonies under a standard FITC filter. Colonies were marked for selection and expansion where appropriate. Following visualization, the basal media was replaced by fresh human iPSC growth media and the selected colonies were either manually picked or returned to the normal culture conditions.

For terminal AP staining, refer to Methods S1.

### Embryoid body formation and differentiation

PSC colonies were detached and reduced to smaller cell clusters using Collagenase IV and light trituration. Cell clusters were plated on untreated Petri dishes and grown in human iPSC media without basic FGF for 4 days with regular media changes. Cell clusters were then transferred to Geltrex®-coated plates for attachment and incubated for 21 days with regular media changes.

### Immunocytochemistry

For live cell staining and flow cytometry, surface marker antibodies were diluted in DMEM/F-12. The unconjugated primary antibodies used in this study were anti-SSEA4 (1∶200), anti-TRA-1-60 (1∶200), and anti-CD44 (2 µg), which were probed with Alexa Fluor® 488- (1∶500) and Alexa Fluor® 594- (1∶500) conjugated secondary antibodies. Pre-conjugated anti-SSEA4-Alexa Fluor® 647 (15 µl per reaction) was also utilized for staining. Washes and visualizations were performed with DMEM/F-12.

The bead-based depletion of CD44-expressing cells following live staining is described in Methods S1, along with the subsequent quantitative polymerase chain reaction (QPCR) analysis.

To test trilineage differentiation potential of 21-day EBs by intracellular immunocytochemistry, cells were fixed with 4% Paraformaldehyde (US Biologicals) and permeabilized using a blocking solution consisting of 5% Normal Goat Serum, 1% BSA, and 0.1% Triton X-100 in dPBS. The primary antibodies against α-Fetoprotein (AFP, 1∶1000), Smooth Muscle Actin (SMA, 1∶50), and β-III Tubulin (βIIITub, 1∶1000) were diluted in the same blocking solution and probed with Alexa Fluor® 594-conjugated secondary antibodies (1∶500). Samples were washed with dPBS.

IgG isotype controls were used to rule out non-specific staining. Images were obtained using a Zeiss Axio Observer.Z1 microscope and Axiovision software. Flow cytometry was performed using the BD FACSCanto™ Flow Cytometry System or the Attune® Acoustic Focusing Cytometer and analyzed using the FlowJo analysis software. Time lapse imaging acquisition and analysis was carried out using the IncuCyte^TM^ FLR Live Content Imaging System (Essen BioScience).

### Microarray analysis

Samples used for the gene expression analysis were BJ fibroblasts (n = 2), H9 ESCs (n = 4), partially reprogrammed iPSCs (n = 2) and fully reprogrammed iPSCs (n = 8) (Table 1). Total RNA was isolated from cells using TRIzol® Reagent, with contaminating genomic DNA eliminated using the DNA-free™ kit. The resulting RNA was assessed for quality by gel electrophoresis and processed for array hybridization to a Human HT-12 v4.0 Beadchip array as suggested by the manufacturer (Illumina).

**Table 1:** Description of parental BJ fibroblasts, control pluripotent H9 ESCs and episomal iPSCs, and iPSC clones used in the study.

Sample #	Sample ID	Cells	Passage #	On feeders/ Feeder-free	Karyotype	Pluripotency markers	PluriTest	Trilineage differentiation
1	BJ	Parental human BJ fibroblasts	P4	N/A	ND	No	Fail	ND
2	BJ P7	Parental human BJ fibroblasts	P7	N/A	ND	No	Fail	ND
3	H9 ESC	H9 ESCs	P53	On feeders	ND	Yes	Pass	Yes
4	ff H9	H9 ESCs	P53	Feeder-free	ND	Yes	Pass	Yes
5	H9 ESC P53	H9 ESCs	P53	On feeders	ND	Yes	Pass	Yes
6	ff H9 ESC P53	H9 ESCs	P53	Feeder-free	ND	Yes	Pass	Yes
7	Ep iPSC	Gibco® Episomal iPSC	P32	On feeders	ND	Yes	Pass	Yes
8	BS1-D P16	iPSC, Clone D	P16	On feeders	**CLG-6664 (p12): NORMAL –46, XY ** [**20**]	Yes	Pass	Yes
9	BS1-F P16	iPSC, Clone F	P16	On feeders	**CLG-6666 (p12): NORMAL –46, XY ** [**19**] **; **46, XY, del(9)(q10) [1]	Yes	Pass	Yes
10	BS1-L P16	iPSC, Clone L	P16	On feeders	**CLG-6665 (p12): NORMAL –46, XY ** [**19**] **; **47, XY, +9 [1]	Yes	Pass	Yes
11	BS3-III P5	iPSC, Clone III	P5	Feeder-free	See next row	Yes	Fail	No
12	BS3-III P16	iPSC, Clone III	P16	Feeder-free	**CLG-8246 (p11): NORMAL –46, XY ** [**20**]	Yes	Pass	Yes
13	BS3-LLL P5	iPSC, Clone LLL	P5	Feeder-free	See next row	Yes	Fail	No
14	BS3-LLL P17	iPSC, Clone LLL	P17	Feeder-free	**CLG-8247 (p11): NORMAL –46, XY ** [**18**] **; **45, XY, -7 [1]; 45, XY, -16 [1]	Yes	Pass	Yes
15	BS3-C P11	iPSC, Clone C	P11	On feeders	**CLG-7998 (p15): NORMAL –46, XY ** [**20**]	Yes	Pass	Yes
16	BS3-C P32	iPSC, Clone C	P32	On feeders	**CLG-9510 (p30): NORMAL –46, XY ** [**18**] **; **47, XY, +12 [1]; 46, XY, add(9)(q34) [1]	Yes	Pass	Yes

ND  =  Not determined.

Hybridized arrays were scanned with an Illumina Bead array reader confocal scanner. Data were analyzed via the web-based PluriTest™ open access software as well as uploaded to GenomeStudio (Illumina) for background subtraction and conversion into a Partek file for data analysis on the Partek® Genomics Suite™ (Partek Incorporated). The differentially expressed genes were obtained using single factor ANOVA model. The three sample categories, namely H9 ESCs, partially reprogrammed iPSCs and fully reprogrammed iPSCs, were compared to the BJ fibroblast samples (taken as control) and significant lists of differentially expressed genes were obtained using a False Discovery Rate cut-off of 0.05 and a fold-change cut-off of 2. The differentially expressed genes were then filtered according cellular location (plasma membrane) using the Ingenuity Pathway Analysis (Ingenuity® Systems). Canonical Wnt/Beta Catenin pathway analysis was also carried out using Ingenuity Pathway Analysis.

Subsets of the gene expression data that were used for graphs were also subjected to ANOVA statistical analysis.

The microarray data discussed in this publication have been deposited in NCBI's Gene Expression Omnibus [26] and are accessible through GEO Series accession number GSE51980 (http://www.ncbi.nlm.nih.gov/geo/query/acc.cgi? acc = GSE51980).

## Results

**Figure 1:**
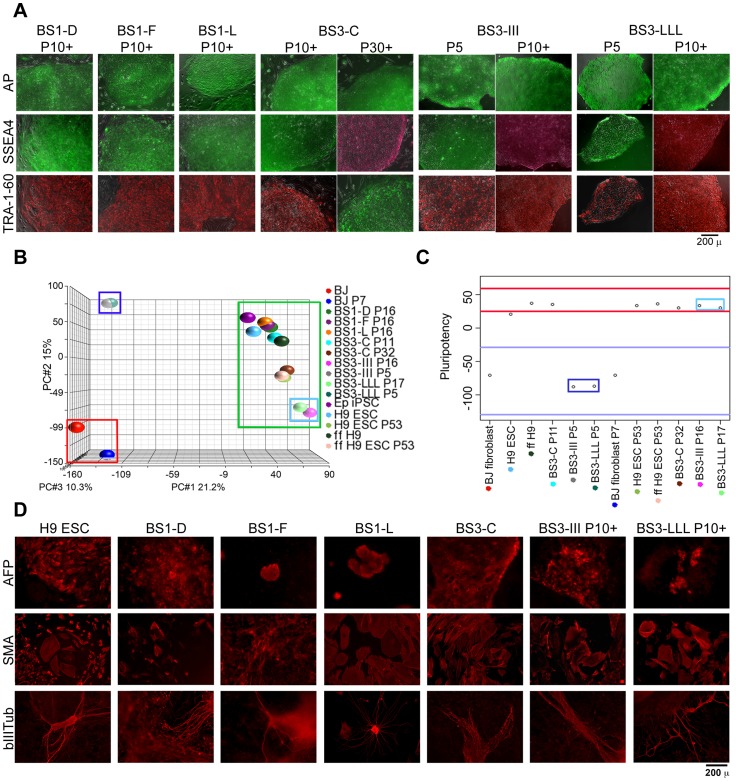
Fully reprogrammed and partially reprogrammed clones are distinguished by combining multiple methods of characterization. (A) iPSC clones generated from BJ fibroblasts and characterized for the presence of pluripotent markers AP, SSEA4 and TRA-1-60. Figure shows phase contrast and fluorescence images merged together (Scale bar: 200 µm). (B) Principal Component Analysis of the global gene expression data from the controls and the iPSCs generated in the study. The three major clusters are demarcated by the red, green, and dark blue boxes. The dark blue and light blue boxes indicate the same clones, but at P5 and P16/17, respectively. (C) Pluripotency scores obtained using PluriTest^TM^ analysis of the global gene expression data for the cells used in the study. The area marked by red lines depicts the region under which 95% of pluripotent samples are expected to fall, while the region between the blue lines depicts where 95% of non-pluripotent samples fall. The dark blue and light blue boxes again indicate the same clones, but at early and late passages, respectively. (D) Immunostaining of trilineage differentiation markers AFP, βIIITub and SMA (red) in Day 21 differentiated ESCs and iPSCs used in the study (Scale bar: 200 µm).

A study was carried out to compare gene expression among 16 samples, including H9 ESCs, parental BJ fibroblasts and newly derived iPSC clones that were grown under different conditions and cultured for varying periods of time. A complete list of the clones and their characteristics are listed in Table 1. Clones were tested to ensure that ≥90% of the cells possessed normal 46, XY karyotypes. The clones were also characterized for the expression of pluripotency markers. The pluripotent markers AP, SSEA4 and TRA-1-60 were found to be uniformly positive and robust in all iPSC clones used in the study (Figure 1A).

Following the preliminary cellular characterization, all samples were subjected to a global transcriptome analysis. The resulting expression data were imported into Partek® Genomics Suite™, log transformed and quantile normalized. The clustering of the samples was determined through Principal Component Analysis (PCA) (Figure 1B). This PCA showed three major clusters, the first represented by the BJ fibroblasts (Figure 1B, red box). The second group included all of the pluripotent clones, namely the H9 ESCs and the iPSC samples at different passage numbers cultured under feeder-dependent or feeder-free conditions (Figure 1B, green box). This group was widely distributed along one dimension away from the BJ fibroblast cluster, suggesting distinct expression from BJ fibroblasts but subtle differences between each other that could have been caused by culture conditions. Interestingly, the third group was comprised of clones BS3-III and BS3-LLL, which were both at passage 5 and expressed pluripotent markers in the preliminary characterization (Figure 1B, dark blue box). This group was distinct from both the parental fibroblasts and the pluripotent cluster. The same clones at higher passage (P>10) cluster with the pluripotent lines (Figure 1B, light blue box).

To independently confirm the pluripotency status of the analyzed cells, the global transcriptome data were also analyzed using the PluriTest^TM^ algorithm [27] (Figure 1C, Table 1). The pluripotency plot for a subset of the samples shows that the negative control BJ fibroblasts have low pluripotency scores. In contrast, all ESCs and most iPSC clones tested have high pluripotency scores and fall within the two red boundaries, which mark the area where greater than 95% of pluripotent cells fall. The two iPSC clones that do not follow this pattern and show a low pluripotency score similar to BJ fibroblasts are BS3-III P5 and BS3-LLL P5 (Figure 1C, dark blue box). These results are consistent with the PCA, where the same early passage samples clustered away from ESCs and established iPSCs. Also consistent with the PCA, the two clones pass the PluriTest when passaged for at least 10 passages (Figure 1C, light blue box).

To further confirm the trilineage differentiation potential of the iPSC clones, the cells were spontaneously differentiated via embryoid body (EB) formation. At the end of 3 weeks of differentiation, they were stained with three lineage-specific antibodies: AFP for endoderm, SMA for mesoderm and βIIITub for ectoderm. Most clones formed EBs and stained positive for the trilineage markers like the H9 ESC positive control, thus functionally confirming their pluripotency. However, clones BS3-III and BS3-LLL failed to form EBs at passage 5 despite exhibiting normal ESC-like morphology. These clones successfully formed EBs and stained positive for trilineage differentiation markers when cultured for at least 10 passages, confirming their functional pluripotency (Figure 1D).

Summarizing the data, most of the iPSC clones expressed pluripotent markers, clustered with ESCs, passed the PluriTest and differentiated into the three germ layers, indicating that they had been fully reprogrammed into pluripotent cells. In contrast, two clones at early passage expressed pluripotency markers but clustered away from ESCs, failed the PluriTest and failed to form EBs. Since these clones exhibited the PSC functional features with additional passaging, the results suggest that the early passage samples were partially reprogrammed.

**Figure 2:**
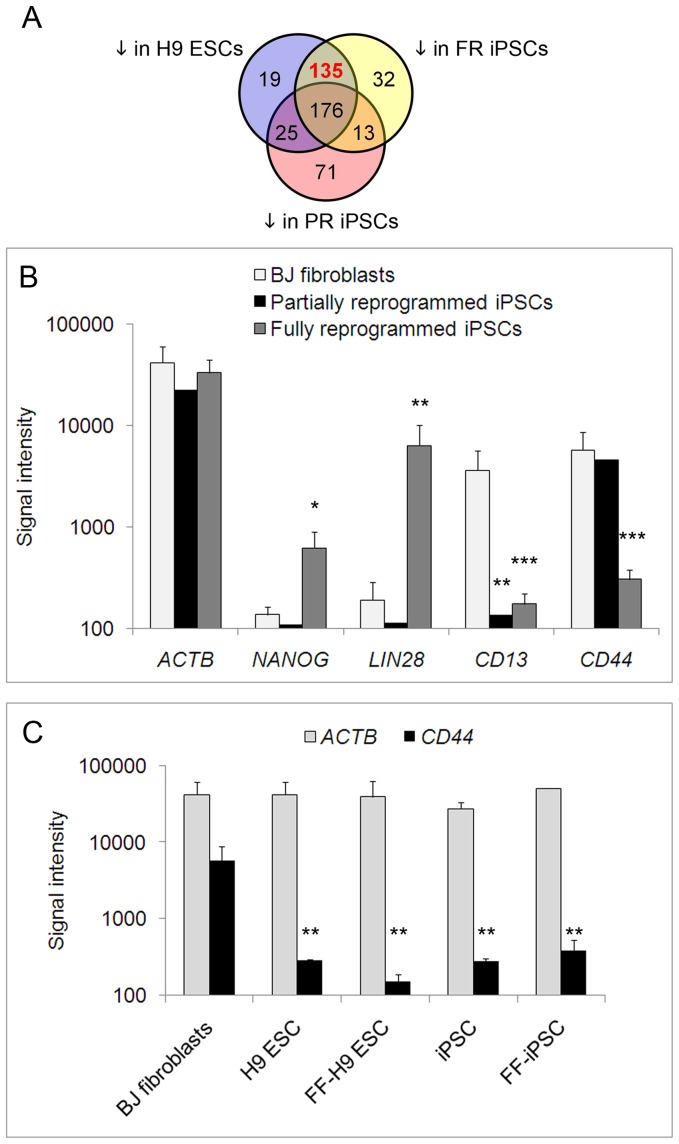
CD44 is differentially expressed during human fibroblast reprogramming. (A) Venn diagram of genes coding for membrane proteins which were downregulated in H9 ESCs, fully reprogrammed iPSCs (FR iPSCs), and/or partially reprogrammed iPSCs (PR iPSCs) compared to BJ fibroblasts according to the global gene expression analysis. (B) Bar graph of microarray signal intensity plotted on the y-axis for each gene for BJ fibroblasts (white bars, n = 2), partially reprogrammed cells (black bars, n = 2) and iPSC clones (gray bars, n = 8). (C) Microarray-detected expression levels of *ACTB* (gray bars) and *CD44* (black bars) plotted on the y-axis for BJ fibroblasts and pluripotent cell types, represented by H9 ESCs cultured with feeders (n = 2), feeder-free (FF) H9 ESCs (n = 2), iPSCs with feeders (n = 6) and feeder-free (FF) iPSCs (n = 2). For the graphs, the error bars represent standard error of the mean. * indicates p-values <0.05, ** marks p-values <0.005, and *** signifies p-values <0.0005 when compared to BJ fibroblasts in an ANOVA analysis.

The above observations imply that pluripotent markers such as AP, SSEA4 and TRA-1-60 are insufficient for distinguishing fully and partially reprogrammed clones and highlight the need for additional surface markers. In order to identify differential markers, the transcriptome of partially reprogrammed clones, fully reprogrammed clones, and ESCs were separately compared to BJ fibroblasts. To specifically find negative surface PSC markers, further analyses of the gene expression data were limited to membrane protein genes that were downregulated compared to BJ fibroblasts. A Venn diagram of the three sets of genes (Figure 2A) revealed that 135 surface markers (Table S1) were significantly down regulated in fully reprogrammed iPSCs and H9 ESCs but not in partially reprogrammed clones. Among the 135 surface markers with the potential to distinguish fully and partially reprogrammed clones, one of the most differentially expressed was the surface glycoprotein, CD44 (Table 2). Interestingly, CD44, a member of the Wnt/Beta Catenin pathway comprising of 175 genes, was one of 53 WNT pathway genes differentially expressed in H9 ESCs compared to fibroblasts as well as one of 58 WNT pathway genes differentially expressed in fully reprogrammed iPSCs (Table S2) [22], [28], [29].

**Table 2:** List of surface markers that are highly downregulated in H9 ESCs and fully reprogrammed cells (FR) compared to BJ fibroblasts but not in partially reprogrammed cells.

Symbol	H9 p-value	H9 fold change	FR p-value	FR fold change	Entrez Gene Name
EMP1	6.43E-05	−52.911	1.38E-04	−27.906	epithelial membrane protein 1
**CD44**	**6.63E-06**	**−46.705**	**2.37E-05**	**−21.839**	**CD44 molecule (Indian blood group)**
PDGFRA	1.92E-08	−45.366	1.11E-08	−38.636	platelet-derived growth factor receptor, alpha polypeptide
PMP22	2.18E-08	−24.084	9.64E-09	−22.694	peripheral myelin protein 22
GNG11	4.10E-04	−21.751	2.98E-04	−18.568	guanine nucleotide binding protein (G protein), gamma 11
HLA-B	6.37E-07	−17.976	2.50E-06	−10.136	major histocompatibility complex, class I, B
RFTN1	2.90E-06	−17.847	3.69E-06	−13.067	raftlin, lipid raft linker 1
NT5E	5.05E-04	−16.631	8.61E-04	−11.007	5′-nucleotidase, ecto (CD73)
C1QTNF5	2.85E-09	−15.146	1.80E-09	−13.228	C1q and tumor necrosis factor related protein 5
B2M	5.71E-07	−14.412	2.83E-07	−13.45	beta-2-microglobulin

In the microarray analysis, CD44 was highly expressed in parental fibroblasts, maintained in partially reprogrammed cells and low in fully reprogrammed iPSCs (Figure 2B). In contrast, the commonly used fibroblast marker CD13 [21], [30] was high in fibroblasts but was low in the partially reprogrammed cells. The PSC markers *NANOG* and *LIN28* were not significantly expressed in parental fibroblasts and in partially reprogrammed cells, but were highly expressed in the reprogrammed iPSCs [31], [32], [33], [34]. The housekeeping gene ACTIN B (ACTB) was expressed evenly across the different samples (Figure 2B). Further comparison of BJ fibroblasts against ESCs and fully reprogrammed iPSCs showed that CD44 was expressed by BJ fibroblasts but not pluripotent stem cells, whether in feeder-dependent or feeder-free conditions (Figure 2C).

**Figure 3:**
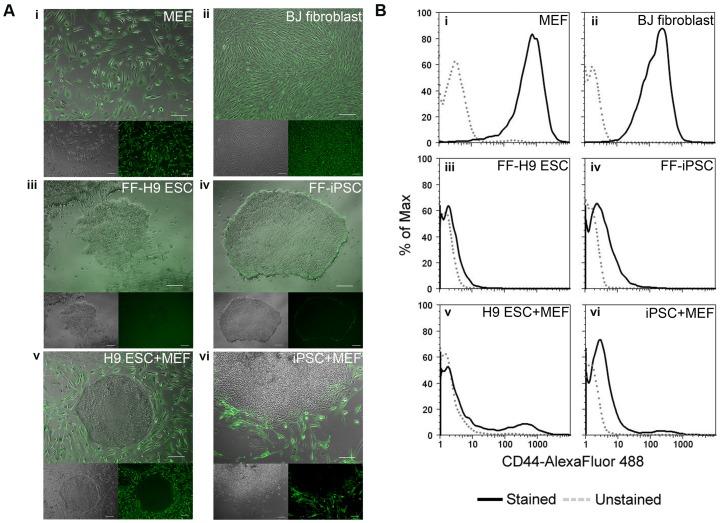
CD44 is a positive fibroblast marker and a negative PSC marker. (A) CD44 immunostaining of (i) MEFs, (ii) BJ fibroblasts, (iii) feeder-free H9 ESCs, (iv) feeder-free iPSCs, (v) H9 ESCs on MEF feeders, and (vi) iPSCs on MEF feeders. The merged images shown consist of phase contrast and CD44 signal (green) (Scale bar: 200 µm). (B) Flow cytometry histograms of CD44-Alexa Fluor® 488 signal intensity in stained samples (solid black line) and unstained samples (dotted gray line) of (i) MEFs, (ii) BJ fibroblasts, (iii) feeder-free H9 ESCs, (iv) feeder-free iPSCs, (v) H9 ESCs on MEF feeders, and (vi) iPSCs on MEF feeders. (FF  =  feeder-free).

Since protein expression can vary from mRNA [35], we confirmed the differential expression pattern of the CD44 protein using indirect immunofluorescence staining on live cells. MEFs and BJ fibroblasts showed robust staining with CD44, while H9 ESCs and established human fibroblast-derived iPSC colonies grown in feeder-free conditions did not show visible staining. In the case of feeder-dependent H9 ESCs and iPSCs, the surrounding MEFs were labeled with CD44 while pluripotent colonies were not (Figure 3A). This pattern was also observed with feeder-dependent iPSCs that were generated through episomal reprogramming [36] and mRNA reprogramming [37] (Figure S1).

To obtain a quantitative measure of CD44 expression in these cells, the stained samples were subjected to flow cytometry analysis. Consistent with the immunostaining results, MEFs and BJ fibroblasts showed a single peak that was significantly shifted to the right compared to the unstained control, hence representing a CD44-expressing population of cells. In contrast, feeder-free H9 ESC and established human iPSC samples resulted in histograms with peaks overlapping the unstained controls, corresponding to the CD44^negative^ cell population. Accordingly, ESCs and iPSCs grown on MEF feeders showed a minor population of CD44^positive^ cells that likely corresponded to the positively stained MEF feeder cells, but the majority of the population was represented by the CD44^negative^ population (Figure 3B).

**Figure 4:**
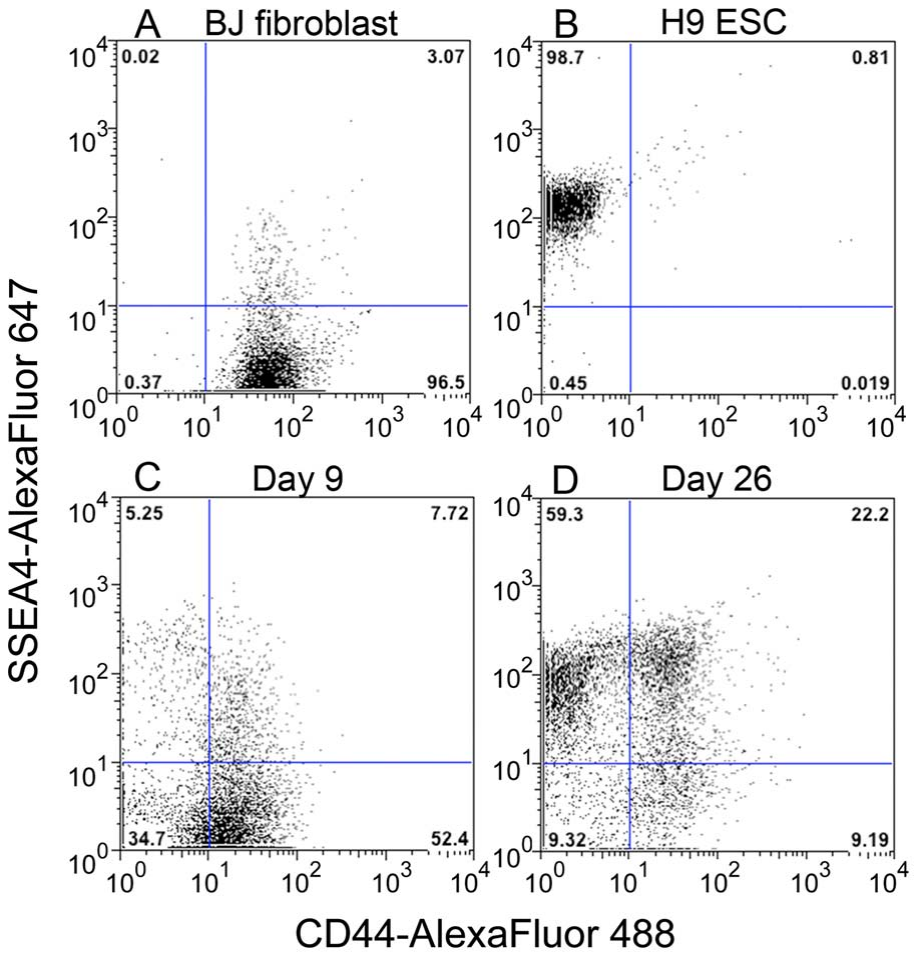
CD44 expression is gradually lost during fibroblast reprogramming. Flow cytometry dot plots with CD44-Alexa Fluor® 488 signal on the x-axis and SSEA4-Alexa Fluor® 647 signal on the y-axis. Lines demarcate quadrants of negative and positive signals for the two fluorophores, and the numbers at each corner indicate the percentage of cells per quadrant. The data compare (A) parental BJ fibroblasts, (B) H9 ESCs, (C) Day 9 reprogramming samples, and (D) Day 26 reprogramming samples.

Since the above results indicate that CD44 is highly expressed in MEFs, parental fibroblasts and partially reprogrammed iPSCs, but is undetectable in fully reprogrammed iPSCs and ESCs, CD44 can function as a negative marker for the identification of pluripotent stem cells. To further investigate the expression pattern of CD44 during the reprogramming process, BJ fibroblasts were transduced with the non-integrating CytoTune®-iPS Sendai Reprogramming Kit [38] and compared to parental BJ fibroblasts and an H9 ESC control. Cells from entire dishes were labeled using antibodies against CD44 and SSEA4, then analyzed using flow cytometry. Figure 4 shows the dot plots for cells expressing CD44 and SSEA4.

BJ fibroblasts were predominantly CD44^positive^ and SSEA4^negative^ (Figure 4A), while the H9 ESCs were CD44^negative^ and SSEA4^positive^ (Figure 4B). At Day 9 post-transduction, the predominant population was CD44^positive^ and SSEA4^negative^, similar to the parental fibroblasts. However, a minor SSEA4^positive^ population, of which a small percentage was CD44^negative^, was observed (Figure 4C). At 26 days post-transduction, when colonies were clearly formed and ready for subcloning, the dot plot showed a less distinct fibroblast-like CD44^positive^ SSEA4^negative^ population, a more prominent double-positive population, and a clear population of CD44^negative^ SSEA4^positive^ reprogrammed cells (Figure 4D).

Taken together, the data demonstrate the gradual loss of CD44 expression as cells are reprogrammed to an ESC-like CD44-negative and SSEA4-expressing phenotype. A detailed time course experiment using CytoTune®-iPS 2.0 Sendai reprogramming kit indicates that progressive loss of CD44 expression and emergence of SSEA4 positive cells begins within the first nine days of reprogramming (Figure S2).

Elimination of CD44^positive^ populations found at Day 26 and subsequent QPCR analysis of the CD44^negative^ cells obtained after depletion revealed a reduced presence of *NR2F1/NR2F2*, *SNAI2*, *RGS4*, and *IL6ST* compared to undepleted cells (Figure S3). These transcripts were confirmed in the microarray analysis to be high in BJ fibroblasts and down regulated in fully reprogrammed iPSCs (Table S3). Additionally, while undepleted reprogramming cultures resulted in AP^positive^ colonies as well as several transformed cells that do not stain for AP (Figure S4A), depletion of CD44 expressing cells eliminated this background resulting in the presence of only AP^positive^ colonies (Figure S4B). CD44 was therefore used as a negative marker during reprogramming, where colonies lacking CD44 expression were easily detected and distinguished from surrounding fibroblasts and CD44-expressing colonies (Figure S5, Video S1). These results highlight the utility of CD44 as a negative marker of pluripotent clones.

**Figure 5:**
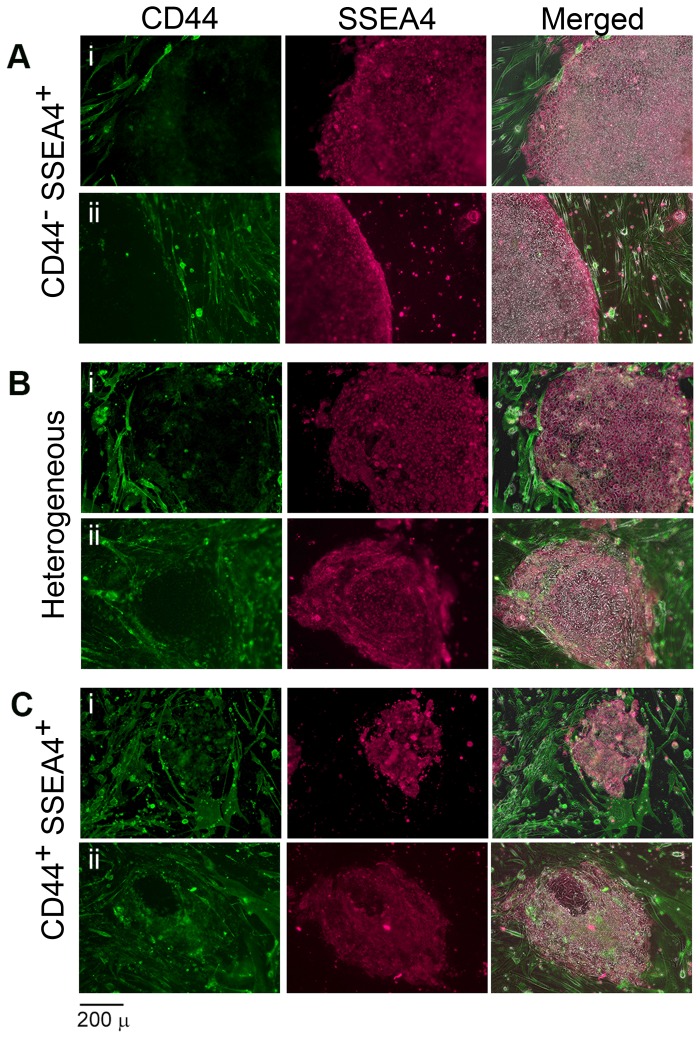
CD44 and SSEA4 co-staining distinguishes three types of colonies. Immunostaining of two independent reprogramming experiments at 23(i) and 26 days (ii) after transduction using antibodies against SSEA4 (magenta), and CD44 (green). The merged panel provides an overlay of the phase contrast image with both fluorescence signals. Both reprogramming experiments yield (A) CD44^negative^ SSEA4^positive^ colonies, (B) heterogeneous colonies, and (C) CD44^positive^ SSEA4^positive^ colonies (Scale bar: 200 µm).

To further utilize negative and positive markers in identifying emerging iPSCs, Day 23 and Day 26 cultures were stained with antibodies against CD44 and SSEA4. Both experiments yielded some colonies with compact cells and defined edges, typical of pluripotent stem cell colonies. These colonies expressed SSEA4 and did not show detectable expression of CD44 (Figure 5A). Other colonies expressed SSEA4 but did not have clear boundaries or contained sections with CD44 expression, suggesting a heterogeneous mixture of cells with varying levels of reprogramming (Figure 5B). A third type of colony expressed CD44 along with SSEA4, indicating that the cells are in an intermediate stage or reprogramming (Figure 5C). Fibroblast-like cells that were positive for CD44 and negative for SSEA4 were also observed outside of these colonies (data not shown). Episomal reprogramming methods show similar clones with these patterns of CD44 and SSEA4 expression (Figure S6).

Collectively, the results indicate that CD44 can be used as a definitive negative marker, either alone or in combination with positive markers, during the identification and selection of iPSC clones.

## Discussion

The establishment of new iPSC lines begins with reprogramming parental somatic cells and sub culturing those that exhibit characteristics of pluripotent cells. Many methods have been developed for identifying these cells, such as observing the formation of colonies and the expression of known PSC markers [14], [15], [16]. Here we have confirmed previous observations that these methods have a limited ability to identify and exclude partially reprogrammed cells [13], [21]. In contrast, a combination of positive and negative marker expression enables earlier identification of true high-quality iPSCs. CD13 is one negative marker that is fibroblast-specific and has successfully been used in fluorescence-assisted cell sorting during reprogramming to eliminate unreprogrammed cells [21]. This approach improved the yield of fully reprogrammed iPSCs, thus showing that the combination of positive and negative iPSC markers has great potential for improving the reprogramming workflow. However, our global transcriptome analysis indicates that CD13 is down regulated early and many partially reprogrammed cells maintaining parental or intermediate characteristics even after CD13 is turned off. Thus there is a need to identify markers that will be maintained longer than CD13.

In this study, we identify CD44 as a marker that is expressed in unreprogrammed fibroblasts and partially reprogrammed clones but not in pluripotent stem cells. We further show the transition of CD44^positive^ fibroblasts to CD44^negative^ SSEA4^positive^ pluripotent cells via intermediate stages during reprogramming. CD44 was then used as a marker to identify iPSC clones based on just negative staining or in combination with SSEA4 as a positive stain for pluripotency. The identification of CD44 as a negative marker for pluripotency is consistent with earlier reports in mouse systems where reprogramming intermediates express CD44 even as a *Nanog*-GFP transgene is activated, indicating the progression of the reprogramming process [12]. The reprogramming intermediate represented by CD44 and SSEA4 double positive cells may offer a similar tool to study the molecular changes during that discrete stage of reprogramming.

While CD44 is expressed in fibroblasts, one of the most commonly used somatic cell sources, CD44 is also known to be expressed in lymphocytes and may serve a similar purpose for improving the blood cell reprogramming work flow [23]. Thus, CD44 may have a broader impact on the reprogramming work flow than what has been directly shown here.

In conclusion, we describe CD44 as a negative marker for pluripotent cells, which can be used alone or in combination with SSEA4 during somatic reprogramming of human fibroblasts. This marker combination presents an improvement over traditional methods for identifying iPSCs for expansion. Moreover, the combination opens doors that will help us understand the mechanisms governing the reprogramming process and develop protocols and tools for the effective application of stem cell technologies.

## Supporting Information

Methods S1 (DOCX)

Figure S1 **CD44 is a negative marker for iPSCs reprogrammed through different protocols.** CD44 immunostaining of iPSCs cultured on MEFs and generated using (A) episomal reprogramming and (B) mRNA reprogramming. The large images merge phase contrast and CD44 signal (green), which are also shown separately in the smaller insets (Scale bar: 200 µm).
(TIF)

Figure S2 **CD44 expression starts decreasing during the first 9 days of reprogramming.** Flow cytometry dot plots of reprogramming samples at (A) Day 0, (B) Day 2, (C) Day 3, (D) Day 4, (E) Day 5, (F) Day 6, (G) Day 7, and (H) Day 9 with CD44-Alexa Fluor® 488 signal on the x-axis and SSEA4-Alexa Fluor® 647 signal on the y-axis.
(TIF)

Figure S3 **CD44^positive^ cell depletion eliminates fibroblast-like cells during reprogramming.** Flow cytometry dot plots with CD44-Alexa Fluor® 488 signal (x-axis) and SSEA4-Alexa Fluor® 647 signal (y-axis). The plots depict cells that were analyzed (A) before and (B) after being depleted of CD44 positive cells at Day 26 after transduction. (C) Bar graph showing the percent change of gene expression between depleted samples (n = 2) and undepleted samples (n = 2), as determined by QPCR. Error bars indicate the standard error of mean. * means p-value <0.05 and ** signifies p-value <0.005 in a one-sample *t*-test.
(TIF)

Figure S4 **CD44^positive^ cell depletion improves the quality of reprogrammed cultures.** Terminal AP staining (red) of Day 23 colonies generated by (A) undepleted controls, or (B) cultures that were depleted of CD44-expressing cells prior to seeding. Images of whole wells are shown against white (ia-iva) and black (ib-ivb) backgrounds to reveal red-stained colonies and white unstained cell clusters (arrows), respectively.
(TIF)

Figure S5 **CD44 can be used as a negative marker to identify iPSC colonies during reprogramming.** Multiple images taken from a single Day 21 culture show CD44 immunofluorescence signal (green) over phase contrast. Arrows indicate CD44-expressing colonies while stars mark CD44 negative colonies (Scale bar: 400 µm).
(TIF)

Figure S6 **CD44 and SSEA4 co-staining distinguishes three types of colonies after episomal reprogramming.** Immunostaining of (A) CD44^negative^ SSEA4^positive^, (B) heterogeneous, and (C) CD44^positive^ SSEA4^positive^ colonies at Day 21 after episomal reprogramming. The merged panel provides an overlay of CD44 (green) and SSEA4 (magenta) fluorescence signals while the phase contrast panel shows colony morphology (Scale bar: 200 µm).
(TIF)

Video S1 **CD44 can be used as a negative marker to identify iPSC colonies during reprogramming.** Time lapse imaging of BJ fibroblasts undergoing episomal-based reprogramming. Cells were seeded on MEFs a week after transfection and imaged under phase contrast every 4 hours for 2 weeks prior to CD44 immunofluorescence (green).
(MP4)

Table S1 **List of surface markers that are downregulated in H9 ESCs and fully reprogrammed iPSCs compared to BJ fibroblasts, but not in partially reprogrammed cells.**
(DOCX)

Table S2 **List of Wnt pathway genes that are differentially expressed in H9 ESCs or fully reprogrammed iPSCs compared to BJ fibroblasts.**
(DOCX)

Table S3 **Selected genes that were differentially expressed in H9 ESCs and fully reprogrammed iPSCs compared to BJ fibroblasts.**
(DOCX)
