# Supplementary material for: CD44 Is a Negative Cell Surface Marker for Pluripotent Stem Cell Identification during Human Fibroblast Reprogramming
Source: PLoS One. 2014 Jan 9;9(1):e85419. doi: 10.1371/journal.pone.0085419 (PMC3887044; doi:10.1371/journal.pone.0085419)
Supplement: Methods S1 — (DOCX) [file pone.0085419.s001.docx]

**Supplementary Methods S1**

*Epi5^TM^ episomal-based reprogramming*: BJ fibroblasts were harvested in single cell suspension and were transfected with the Epi5™ Episomal iPSC reprogramming kit as per the manufacturer’s directions using the Neon® electroporation kit. Transfected cells were seeded directly at the desired densities onto MEF feeders in fibroblast media overnight. The transfected cells then were switched to N2/B-27 media comprised of DMEM/F-12, N-2 supplement, B-27® supplement, 10mM MEM-NEAA solution, 100 mM 2-Mercaptoethanol and 10 ng/ml basic FGF. Cultures were maintained on N2/B-27 media until day 15 post transfection, at which time the media was switched to human iPSC media until cultures were ready for selection.

*mRNA-based reprogramming*: BJ fibroblasts were seeded onto mitotically inactivated Newborn Human Foreskin Fibroblasts (NuFF) purchased from Global Stem in Pluriton™ media (Stemgent). The cells were transfected for 17 consecutive days with the Stemgent® mRNA Reprogramming System (Stemgent) and Lipofectamine® RNAiMAX transfection reagents according to the manufacturer’s instructions. Colonies at 21 to 25 days post-transfection were identified and scored using a 27-gauge needle, picked manually and expanded further on MEFs using human iPSC media supplemented with 10 ng/mL bFGF.

*CD44^positive^ cell depletion:* Reprogramming cultures were harvested in single cell suspension using TrypLE™ Express and incubated with biotin-conjugated rat anti-CD44 antibody (1 ug/1x10^6^ target cells, BD Pharmingen^TM^). The cell suspension was then incubated with Dynabeads® Biotin Binder streptavidin-coated magnetic beads according to the manufacturer’s protocol. CD44-expressing cells that were bound by the biotin-conjugated antibody were captured by the magnetic beads and pulled down with the DynaMag™ magnet. The supernatant was then either reseeded onto MEF-coated dishes with human iPSC media, probed with anti-SSEA4 and CD44 antibodies for FACS analysis as previously described, or harvested for RNA isolation.

*Terminal AP Staining.* Reprogrammed colonies were gauged on the master reprogramming plates by using the colorimetric Vector® Red Alkaline Phosphatase kit (Vector Labs) as per the manufacturer’s manual.

*Quantitative Polymerase Chain Reaction (QPCR) Analysis:* RNA from all cultures was extracted with TRIzol® Reagent. In preparation for QPCR, the RNA was converted to cDNA using the High Capacity cDNA Reverse Transcription Kit. QPCR of the cDNA was performed on a StepOnePlus™ Real-Time PCR System with the standard TaqMan® amplification protocol and the following TaqMan® assays: ACTB (Hs01060665_g1, Hs99999903_m1), NR2F1/NR2F2 (Hs01354342_mH), SNAI2 (Hs00950344_m1), RGS4 (Hs01111690_g1), IL6ST (Hs00174360_m1), and NANOG (Hs02387400_g1). Statistical significance of the percent changes in gene expression was determined through one-sample *t*-tests against zero with n=2.
