# Supplementary figures and images for: CD44 Is a Negative Cell Surface Marker for Pluripotent Stem Cell Identification during Human Fibroblast Reprogramming
Source: PLoS One. 2014 Jan 9;9(1):e85419. doi: 10.1371/journal.pone.0085419 (PMC3887044; doi:10.1371/journal.pone.0085419)

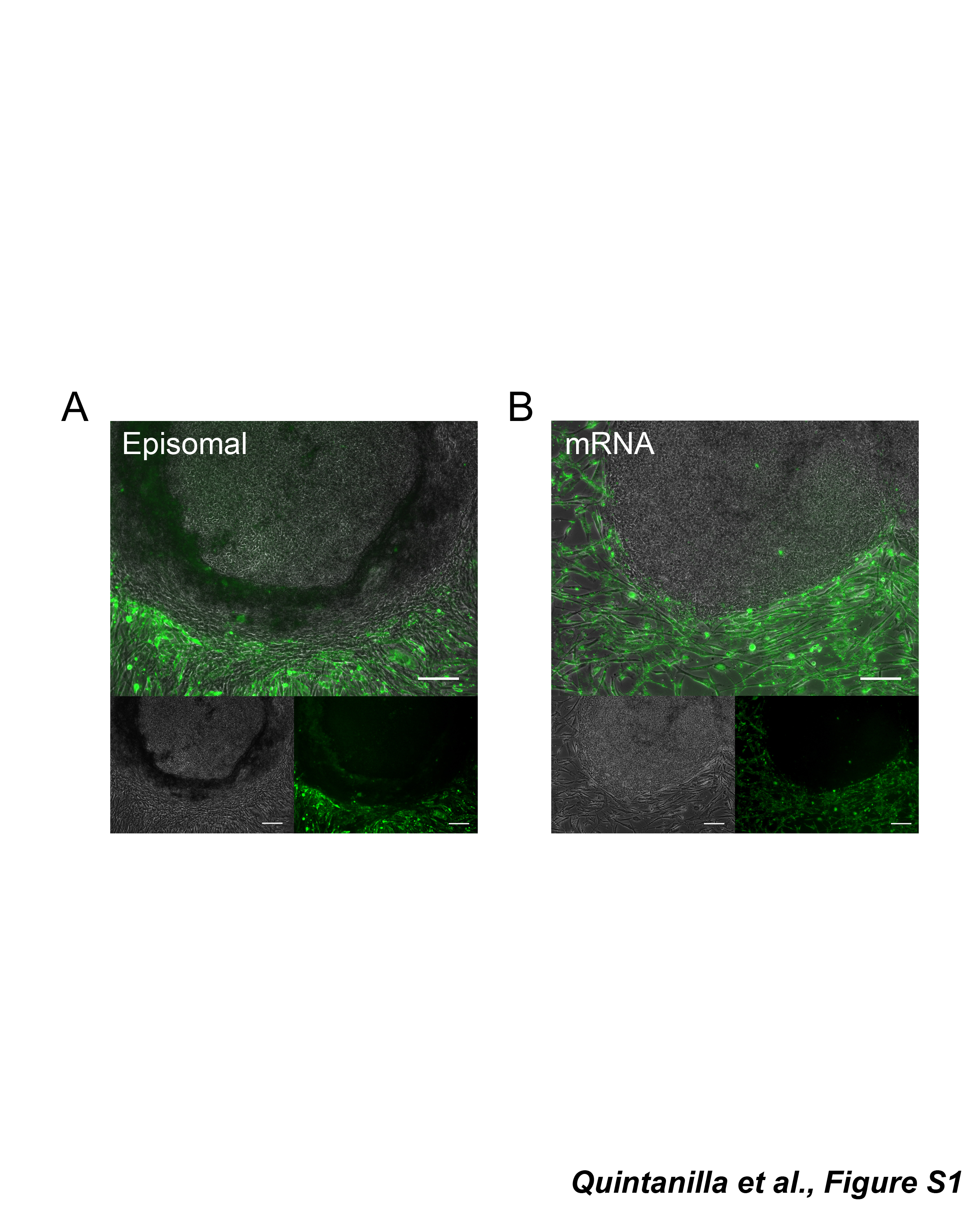

Supplement: Figure S1 — CD44 is a negative marker for iPSCs reprogrammed through different protocols. CD44 immunostaining of iPSCs cultured on MEFs and generated using (A) episomal reprogramming and (B) mRNA reprogramming. The large images merge phase contrast and CD44 signal (green), which are also shown separately in the smaller insets (Scale bar: 200 µm). (TIF) [file pone.0085419.s002.tif]

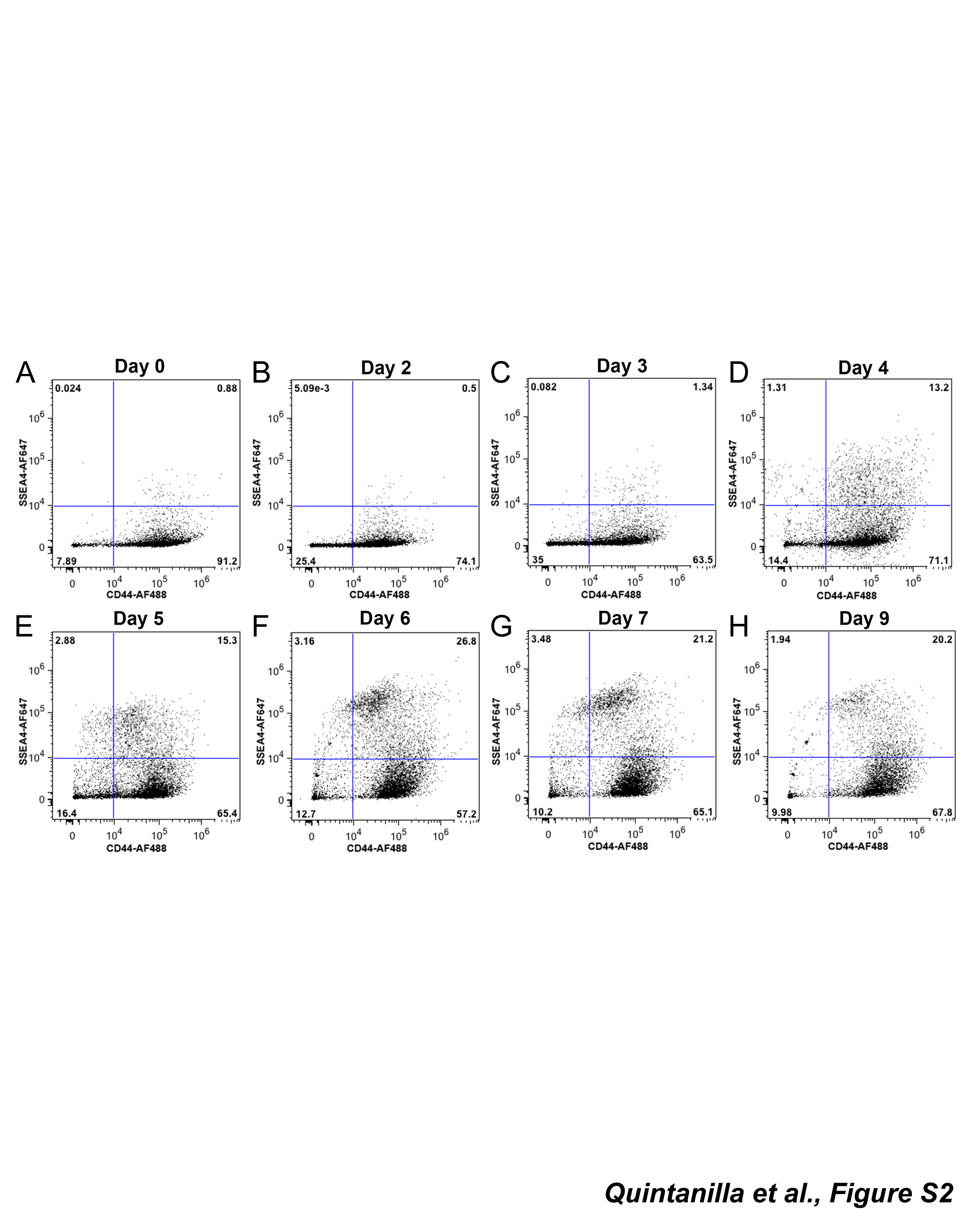

Supplement: Figure S2 — CD44 expression starts decreasing during the first 9 days of reprogramming. Flow cytometry dot plots of reprogramming samples at (A) Day 0, (B) Day 2, (C) Day 3, (D) Day 4, (E) Day 5, (F) Day 6, (G) Day 7, and (H) Day 9 with CD44-Alexa Fluor® 488 signal on the x-axis and SSEA4-Alexa Fluor® 647 signal on the y-axis. (TIF) [file pone.0085419.s003.tif]

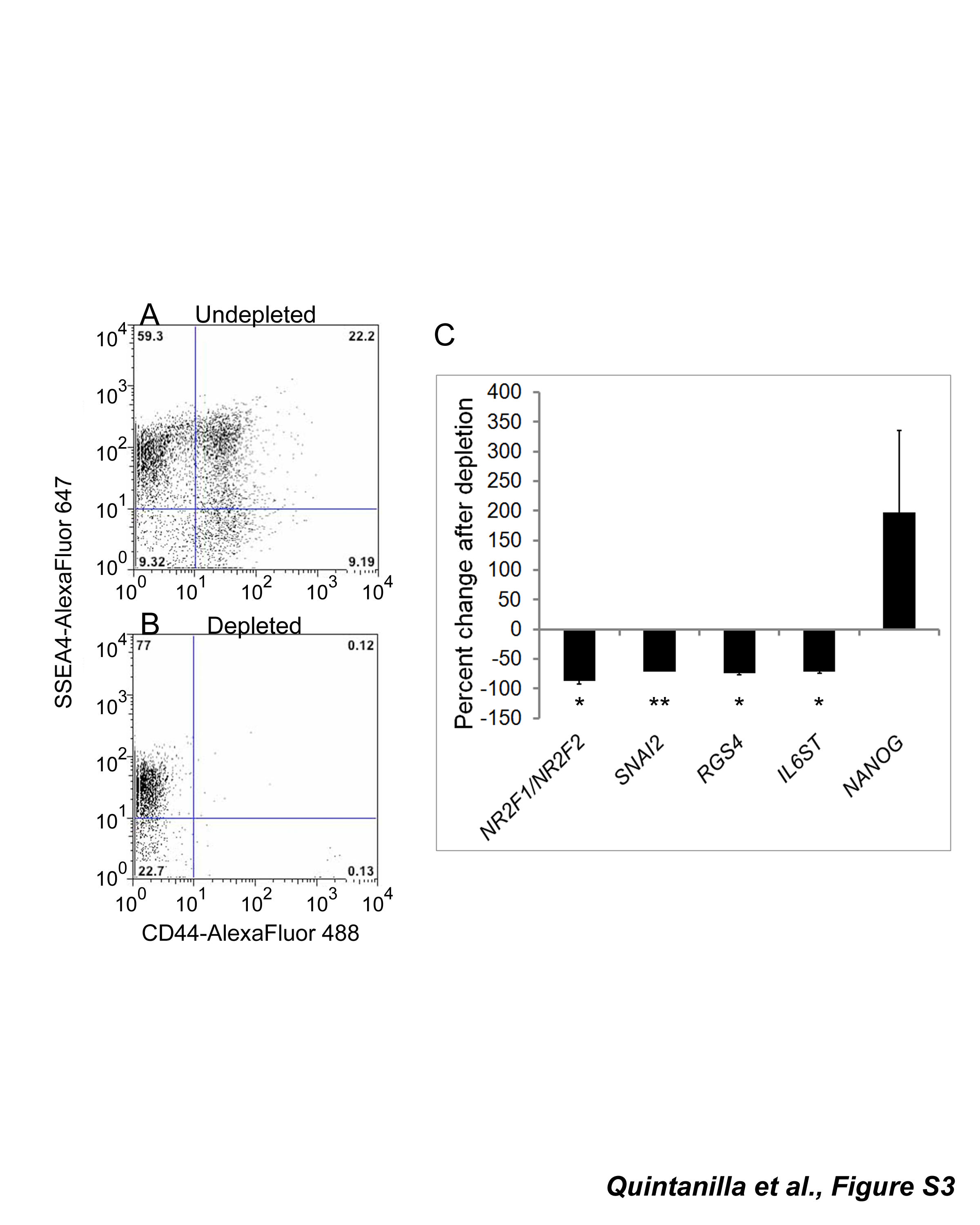

Supplement: Figure S3 — CD44positive cell depletion eliminates fibroblast-like cells during reprogramming. Flow cytometry dot plots with CD44-Alexa Fluor® 488 signal (x-axis) and SSEA4-Alexa Fluor® 647 signal (y-axis). The plots depict cells that were analyzed (A) before and (B) after being depleted of CD44 positive cells at Day 26 after transduction. (C) Bar graph showing the percent change of gene expression between depleted samples (n = 2) and undepleted samples (n = 2), as determined by QPCR. Error bars indicate the standard error of mean. * means p-value <0.05 and ** signifies p-value <0.005 in a one-sample t-test. (TIF) [file pone.0085419.s004.tif]

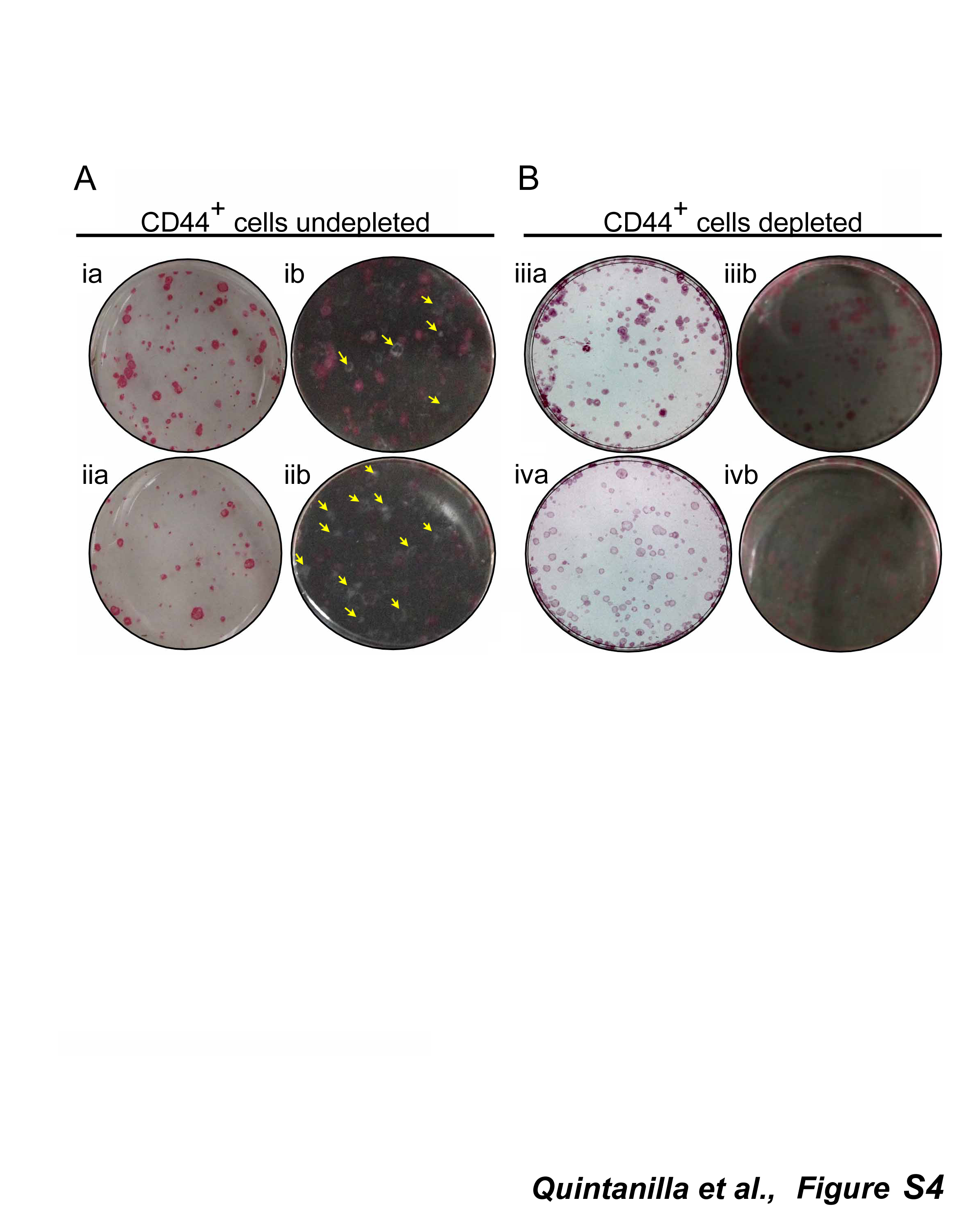

Supplement: Figure S4 — CD44positive cell depletion improves the quality of reprogrammed cultures. Terminal AP staining (red) of Day 23 colonies generated by (A) undepleted controls, or (B) cultures that were depleted of CD44-expressing cells prior to seeding. Images of whole wells are shown against white (ia-iva) and black (ib-ivb) backgrounds to reveal red-stained colonies and white unstained cell clusters (arrows), respectively. (TIF) [file pone.0085419.s005.tif]

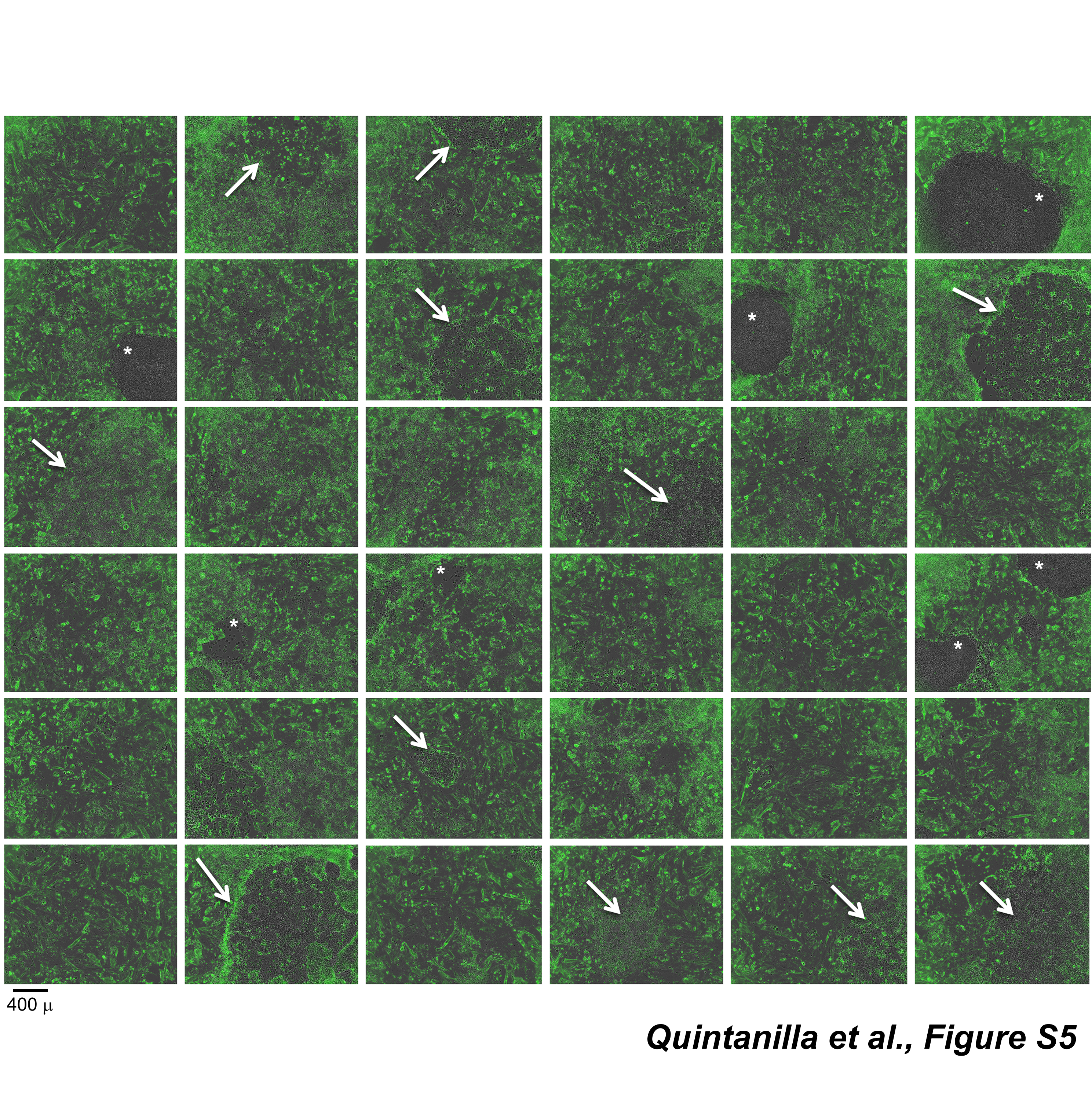

Supplement: Figure S5 — CD44 can be used as a negative marker to identify iPSC colonies during reprogramming. Multiple images taken from a single Day 21 culture show CD44 immunofluorescence signal (green) over phase contrast. Arrows indicate CD44-expressing colonies while stars mark CD44 negative colonies (Scale bar: 400 µm). (TIF) [file pone.0085419.s006.tif]

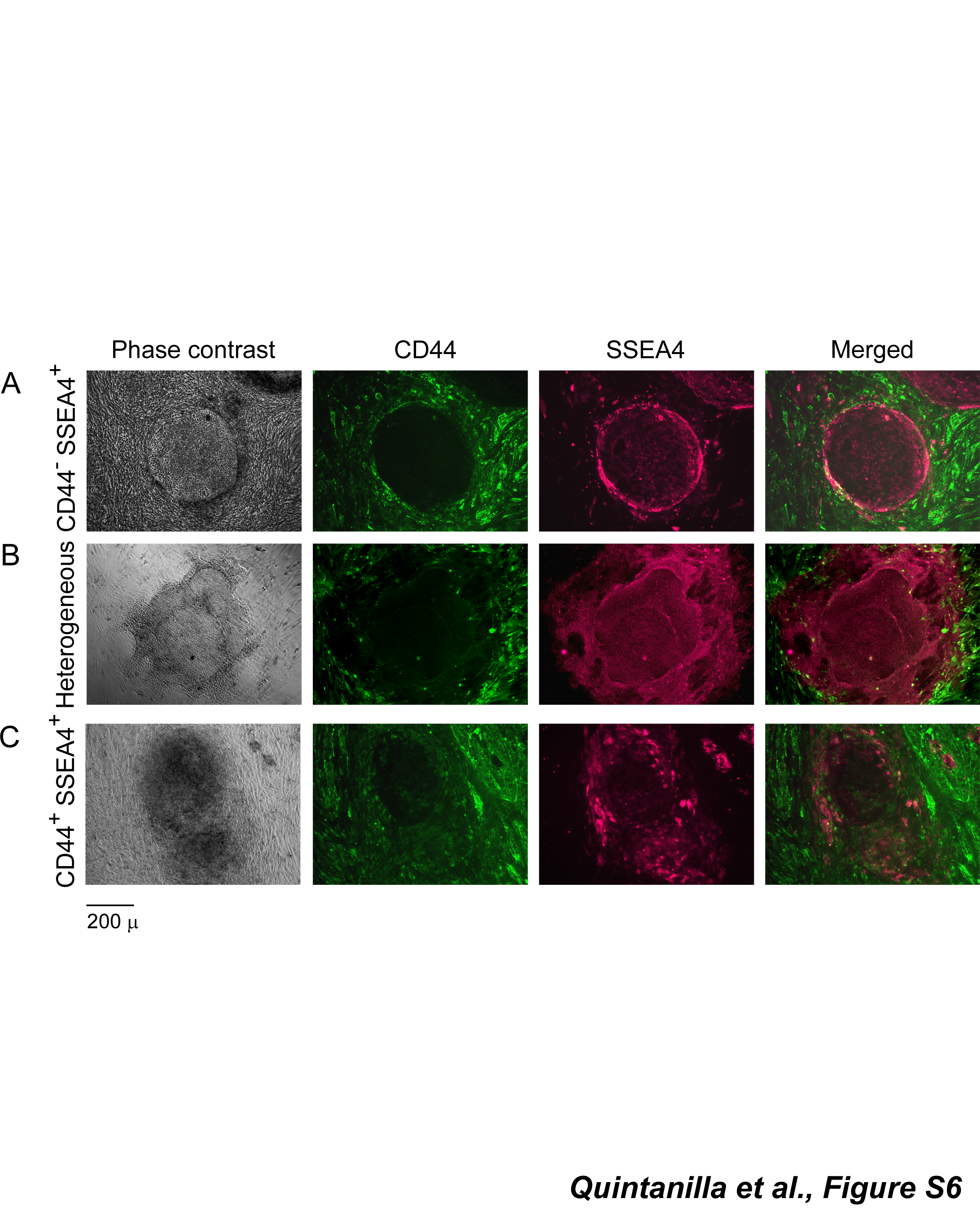

Supplement: Figure S6 — CD44 and SSEA4 co-staining distinguishes three types of colonies after episomal reprogramming. Immunostaining of (A) CD44negative SSEA4positive, (B) heterogeneous, and (C) CD44positive SSEA4positive colonies at Day 21 after episomal reprogramming. The merged panel provides an overlay of CD44 (green) and SSEA4 (magenta) fluorescence signals while the phase contrast panel shows colony morphology (Scale bar: 200 µm). (TIF) [file pone.0085419.s007.tif]
