# Supplementary material for: CD44 Is a Negative Cell Surface Marker for Pluripotent Stem Cell Identification during Human Fibroblast Reprogramming
Source: PLoS One. 2014 Jan 9;9(1):e85419. doi: 10.1371/journal.pone.0085419 (PMC3887044; doi:10.1371/journal.pone.0085419)
Supplement: Table S1 — List of surface markers that are downregulated in H9 ESCs and fully reprogrammed iPSCs compared to BJ fibroblasts, but not in partially reprogrammed cells. (DOCX) [file pone.0085419.s009.docx]

**Table S1:** List of surface markers that are downregulated in H9 ESCs and fully reprogrammed iPSCs compared to BJ fibroblasts, but not in partially reprogrammed cells.

| **Symbol** | **H9 p-value** | **H9 fold change** | **FR p-value** | **FR fold change** | **Entrez Gene Name** | **Type(s)** |
| --- | --- | --- | --- | --- | --- | --- |
| EMP1 | 6.43E-05 | -52.911 | 1.38E-04 | -27.906 | epithelial membrane protein 1 | other |
| **CD44** | **6.63E-06** | **-46.705** | **2.37E-05** | **-21.839** | **CD44 molecule (Indian blood group)** | **enzyme** |
| PDGFRA | 1.92E-08 | -45.366 | 1.11E-08 | -38.636 | platelet-derived growth factor receptor, alpha polypeptide | kinase |
| PMP22 | 2.18E-08 | -24.084 | 9.64E-09 | -22.694 | peripheral myelin protein 22 | other |
| GNG11 | 4.10E-04 | -21.751 | 2.98E-04 | -18.568 | guanine nucleotide binding protein (G protein), gamma 11 | enzyme |
| HLA-B | 6.37E-07 | -17.976 | 2.50E-06 | -10.136 | major histocompatibility complex, class I, B | transmembrane receptor |
| RFTN1 | 2.90E-06 | -17.847 | 3.69E-06 | -13.067 | raftlin, lipid raft linker 1 | other |
| NT5E | 5.05E-04 | -16.631 | 8.61E-04 | -11.007 | 5'-nucleotidase, ecto (CD73) | phosphatase |
| C1QTNF5 | 2.85E-09 | -15.146 | 1.80E-09 | -13.228 | C1q and tumor necrosis factor related protein 5 | transmembrane receptor |
| B2M | 5.71E-07 | -14.412 | 2.83E-07 | -13.45 | beta-2-microglobulin | transmembrane receptor |
| SEMA5A | 1.20E-10 | -13.063 | 2.26E-11 | -14.941 | sema domain, seven thrombospondin repeats (type 1 and type 1-like), transmembrane domain (TM) and short cytoplasmic domain, (semaphorin) 5A | transmembrane receptor |
| HEG1 | 8.27E-07 | -12.726 | 6.14E-07 | -10.893 | heart development protein with EGF-like domains 1 | other |
| EMP3 | 4.64E-04 | -10.467 | 5.69E-04 | -8.084 | epithelial membrane protein 3 | other |
| ANTXR2 | 5.31E-06 | -10.261 | 1.61E-06 | -10.867 | anthrax toxin receptor 2 | transmembrane receptor |
| BMPR2 | 1.40E-05 | -9.604 | 1.98E-05 | -7.332 | bone morphogenetic protein receptor, type II (serine/threonine kinase) | kinase |
| OSMR | 4.26E-06 | -8.676 | 3.32E-06 | -7.545 | oncostatin M receptor | transmembrane receptor |
| GNG12 | 3.84E-06 | -8.008 | 4.32E-06 | -6.537 | guanine nucleotide binding protein (G protein), gamma 12 | enzyme |
| IFNAR1 | 6.25E-07 | -7.677 | 7.57E-07 | -6.219 | interferon (alpha, beta and omega) receptor 1 | transmembrane receptor |
| KCNH1 | 4.30E-07 | -7.649 | 7.10E-07 | -5.885 | potassium voltage-gated channel, subfamily H (eag-related), member 1 | ion channel |
| EPS8 | 2.45E-07 | -7.03 | 1.10E-07 | -6.786 | epidermal growth factor receptor pathway substrate 8 | peptidase |
| SIRPA | 1.37E-07 | -6.813 | 1.40E-08 | -8.585 | signal-regulatory protein alpha | phosphatase |
| HLA-F | 5.40E-06 | -6.492 | 6.35E-05 | -3.759 | major histocompatibility complex, class I, F | transmembrane receptor |
| ANXA4 | 3.38E-06 | -6.257 | 2.69E-06 | -5.539 | annexin A4 | other |
| GLRB | 2.90E-06 | -6.126 | 1.05E-06 | -6.198 | glycine receptor, beta | ion channel |
| MRC2 | 4.77E-08 | -5.917 | 2.05E-08 | -5.752 | mannose receptor, C type 2 | transmembrane receptor |
| CD99L2 | 7.07E-06 | -5.895 | 8.03E-07 | -7.375 | CD99 molecule-like 2 | other |
| F3 | 3.92E-08 | -5.737 | 1.93E-07 | -3.977 | coagulation factor III (thromboplastin, tissue factor) | transmembrane receptor |
| CD59 | 2.32E-08 | -5.648 | 1.11E-08 | -5.401 | CD59 molecule, complement regulatory protein | other |
| GPC1 | 9.62E-08 | -5.6 | 3.23E-08 | -5.668 | glypican 1 | transmembrane receptor |
| AXL | 6.46E-04 | -5.534 | 8.65E-04 | -4.503 | AXL receptor tyrosine kinase | kinase |
| SSFA2 | 1.75E-06 | -5.493 | 1.55E-06 | -4.822 | sperm specific antigen 2 | other |
| TRAK2 | 1.50E-05 | -5.402 | 4.01E-05 | -4.013 | trafficking protein, kinesin binding 2 | transporter |
| CD151 | 3.76E-07 | -5.384 | 9.95E-07 | -4.068 | CD151 molecule (Raph blood group) | other |
| GRK5 | 6.82E-08 | -5.375 | 1.87E-08 | -5.606 | G protein-coupled receptor kinase 5 | kinase |
| PAM | 6.97E-04 | -5.262 | 1.60E-03 | -3.888 | peptidylglycine alpha-amidating monooxygenase | enzyme |
| TNFAIP1 | 1.67E-09 | -5.247 | 2.42E-10 | -5.979 | tumor necrosis factor, alpha-induced protein 1 (endothelial) | ion channel |
| TBC1D9 | 7.96E-04 | -5.151 | 2.13E-04 | -5.801 | TBC1 domain family, member 9 (with GRAM domain) | other |
| FAS | 8.34E-06 | -5.09 | 1.80E-05 | -3.952 | Fas cell surface death receptor | transmembrane receptor |
| RHOC | 7.53E-06 | -5.025 | 3.27E-06 | -4.951 | ras homolog family member C | enzyme |
| MOK | 5.97E-05 | -4.847 | 4.03E-05 | -4.494 | MOK protein kinase | kinase |
| CD47 | 3.26E-07 | -4.795 | 7.03E-08 | -5.185 | CD47 molecule | transmembrane receptor |
| RGMB | 5.31E-05 | -4.773 | 2.63E-05 | -4.653 | RGM domain family, member B | other |
| ITGA3 | 1.59E-04 | -4.753 | 9.14E-04 | -3.16 | integrin, alpha 3 (antigen CD49C, alpha 3 subunit of VLA-3 receptor) | other |
| CD99 | 9.51E-04 | -4.585 | 3.49E-05 | -7.7 | CD99 molecule | other |
| BTN3A2 | 3.66E-08 | -4.563 | 1.21E-08 | -4.612 | butyrophilin, subfamily 3, member A2 | other |
| IL13RA1 | 7.62E-04 | -4.362 | 2.43E-04 | -4.695 | interleukin 13 receptor, alpha 1 | transmembrane receptor |
| PALLD | 4.94E-03 | -4.346 | 3.33E-03 | -4.157 | palladin, cytoskeletal associated protein | other |
| GEM | 5.24E-08 | -4.12 | 3.77E-09 | -5.112 | GTP binding protein overexpressed in skeletal muscle | enzyme |
| SCARF2 | 7.51E-06 | -4.09 | 3.18E-06 | -4.052 | scavenger receptor class F, member 2 | transmembrane receptor |
| RAB40B | 2.63E-05 | -4.053 | 2.91E-05 | -3.539 | RAB40B, member RAS oncogene family | enzyme |
| LAMP2 | 9.41E-05 | -3.973 | 1.10E-04 | -3.447 | lysosomal-associated membrane protein 2 | enzyme |
| BTN3A3 | 6.36E-04 | -3.893 | 6.12E-05 | -5.1 | butyrophilin, subfamily 3, member A3 | other |
| SARM1 | 5.24E-05 | -3.864 | 3.31E-05 | -3.653 | sterile alpha and TIR motif containing 1 | transmembrane receptor |
| ITGB1 | 1.56E-04 | -3.797 | 7.74E-04 | -2.728 | integrin, beta 1 (fibronectin receptor, beta polypeptide, antigen CD29 includes MDF2, MSK12) | transmembrane receptor |
| PPAP2B | 9.16E-06 | -3.795 | 1.19E-05 | -3.274 | phosphatidic acid phosphatase type 2B | phosphatase |
| KCNJ8 | 6.37E-09 | -3.761 | 1.57E-08 | -3.055 | potassium inwardly-rectifying channel, subfamily J, member 8 | ion channel |
| STIM1 | 1.73E-05 | -3.741 | 1.16E-06 | -4.812 | stromal interaction molecule 1 | ion channel |
| RAMP1 | 6.05E-06 | -3.72 | 1.78E-05 | -2.931 | receptor (G protein-coupled) activity modifying protein 1 | transporter |
| CHIC2 | 2.49E-05 | -3.698 | 2.75E-05 | -3.258 | cysteine-rich hydrophobic domain 2 | other |
| PKD1 | 4.45E-05 | -3.696 | 2.52E-05 | -3.552 | polycystic kidney disease 1 (autosomal dominant) | ion channel |
| PLXNB2 | 2.63E-04 | -3.657 | 2.90E-04 | -3.222 | plexin B2 | transmembrane receptor |
| NLGN1 | 1.10E-03 | -3.606 | 3.98E-04 | -3.79 | neuroligin 1 | enzyme |
| RAB7L1 | 1.06E-05 | -3.603 | 1.95E-06 | -3.985 | RAB7, member RAS oncogene family-like 1 | enzyme |
| SLC31A2 | 2.54E-04 | -3.575 | 2.69E-04 | -3.174 | solute carrier family 31 (copper transporters), member 2 | transporter |
| SLC16A4 | 8.36E-08 | -3.559 | 4.09E-08 | -3.441 | solute carrier family 16, member 4 (monocarboxylic acid transporter 5) | transporter |
| VAMP3 | 1.22E-05 | -3.536 | 4.46E-06 | -3.578 | vesicle-associated membrane protein 3 | other |
| SLC20A2 | 1.90E-04 | -3.531 | 1.57E-03 | -2.422 | solute carrier family 20 (phosphate transporter), member 2 | transporter |
| SGCE | 8.77E-06 | -3.522 | 2.15E-06 | -3.742 | sarcoglycan, epsilon | other |
| TANC1 | 5.99E-04 | -3.516 | 1.47E-04 | -3.884 | tetratricopeptide repeat, ankyrin repeat and coiled-coil containing 1 | other |
| ITGB7 | 8.09E-06 | -3.496 | 9.27E-06 | -3.087 | integrin, beta 7 | transmembrane receptor |
| SNTB1 | 6.59E-04 | -3.48 | 5.86E-03 | -2.305 | syntrophin, beta 1 (dystrophin-associated protein A1, 59kDa, basic component 1) | other |
| PTK7 | 2.66E-03 | -3.465 | 3.56E-03 | -2.964 | protein tyrosine kinase 7 | kinase |
| IFIT5 | 1.86E-06 | -3.322 | 1.11E-06 | -3.161 | interferon-induced protein with tetratricopeptide repeats 5 | other |
| SERINC1 | 7.75E-04 | -3.318 | 1.89E-04 | -3.668 | serine incorporator 1 | transporter |
| ZYX | 8.68E-04 | -3.183 | 1.37E-03 | -2.706 | zyxin | other |
| TMEM59 | 3.68E-06 | -3.083 | 1.09E-05 | -2.517 | transmembrane protein 59 | peptidase |
| ADAM9 | 2.62E-03 | -3.079 | 8.13E-07 | -4.919 | ADAM metallopeptidase domain 9 | peptidase |
| CLTB | 1.19E-03 | -3.074 | 6.27E-04 | -3.045 | clathrin, light chain B | other |
| FAM127A | 2.20E-05 | -3.055 | 2.23E-06 | -3.6 | family with sequence similarity 127, member A | other |
| ITGB1BP1 | 4.47E-07 | -3.034 | 1.37E-07 | -3.095 | integrin beta 1 binding protein 1 | other |
| BOC | 3.35E-03 | -3.028 | 3.36E-04 | -3.946 | BOC cell adhesion associated, oncogene regulated | other |
| ILK | 1.73E-05 | -3.022 | 1.68E-05 | -2.753 | integrin-linked kinase | kinase |
| PLXNA3 | 7.40E-04 | -3.005 | 2.55E-04 | -3.141 | plexin A3 | transmembrane receptor |
| GPR137B | 1.21E-03 | -2.917 | 5.64E-04 | -2.94 | G protein-coupled receptor 137B | other |
| SLC12A4 | 2.51E-03 | -2.899 | 1.65E-03 | -2.802 | solute carrier family 12 (potassium/chloride transporters), member 4 | transporter |
| NRSN2 | 1.70E-05 | -2.894 | 1.92E-06 | -3.332 | neurensin 2 | other |
| MRAS | 1.33E-06 | -2.874 | 9.34E-07 | -2.709 | muscle RAS oncogene homolog | enzyme |
| CD63 | 5.20E-04 | -2.856 | 6.24E-04 | -2.551 | CD63 molecule | other |
| AMFR | 5.34E-06 | -2.765 | 1.10E-06 | -2.948 | autocrine motility factor receptor, E3 ubiquitin protein ligase | transmembrane receptor |
| CAP2 | 1.37E-03 | -2.734 | 2.86E-05 | -4.243 | CAP, adenylate cyclase-associated protein, 2 (yeast) | other |
| CD58 | 1.24E-05 | -2.724 | 2.02E-05 | -2.388 | CD58 molecule | transmembrane receptor |
| PLA2G4C | 1.38E-03 | -2.669 | 3.10E-04 | -2.952 | phospholipase A2, group IVC (cytosolic, calcium-independent) | enzyme |
| SGCB | 3.12E-06 | -2.669 | 3.50E-06 | -2.426 | sarcoglycan, beta (43kDa dystrophin-associated glycoprotein) | other |
| ANO6 | 1.57E-03 | -2.657 | 2.31E-03 | -2.328 | anoctamin 6 | ion channel |
| ITFG1 | 1.98E-03 | -2.654 | 7.12E-04 | -2.779 | integrin alpha FG-GAP repeat containing 1 | other |
| GNA11 | 1.69E-05 | -2.65 | 4.13E-05 | -2.248 | guanine nucleotide binding protein (G protein), alpha 11 (Gq class) | enzyme |
| ATP7A | 1.02E-04 | -2.608 | 1.80E-04 | -2.272 | ATPase, Cu++ transporting, alpha polypeptide | transporter |
| KCNJ2 | 7.17E-04 | -2.608 | 3.14E-04 | -2.635 | potassium inwardly-rectifying channel, subfamily J, member 2 | ion channel |
| ASAP1 | 5.78E-06 | -2.587 | 1.91E-07 | -3.316 | ArfGAP with SH3 domain, ankyrin repeat and PH domain 1 | other |
| INPP5A | 1.90E-03 | -2.544 | 4.58E-04 | -2.792 | inositol polyphosphate-5-phosphatase, 40kDa | phosphatase |
| FADS3 | 9.13E-06 | -2.495 | 1.43E-05 | -2.221 | fatty acid desaturase 3 | enzyme |
| MARCKS | 1.67E-03 | -2.484 | 3.28E-04 | -2.784 | myristoylated alanine-rich protein kinase C substrate | other |
| ARRDC3 | 1.42E-04 | -2.474 | 6.50E-05 | -2.465 | arrestin domain containing 3 | other |
| RGS11 | 7.79E-08 | -2.473 | 1.33E-08 | -2.633 | regulator of G-protein signaling 11 | enzyme |
| ITGAE | 7.76E-04 | -2.454 | 1.09E-03 | -2.191 | integrin, alpha E (antigen CD103, human mucosal lymphocyte antigen 1; alpha polypeptide) | other |
| RASA3 | 3.26E-07 | -2.45 | 1.47E-06 | -2.035 | RAS p21 protein activator 3 | ion channel |
| ADAM15 | 8.05E-04 | -2.448 | 1.21E-03 | -2.17 | ADAM metallopeptidase domain 15 | peptidase |
| FLOT1 | 5.48E-04 | -2.44 | 5.00E-05 | -2.923 | flotillin 1 | other |
| KLHL20 | 7.12E-06 | -2.431 | 1.83E-05 | -2.09 | kelch-like family member 20 | enzyme |
| HRH1 | 3.14E-05 | -2.428 | 3.71E-05 | -2.215 | histamine receptor H1 | G-protein coupled receptor |
| TSPAN9 | 1.93E-04 | -2.427 | 3.95E-04 | -2.103 | tetraspanin 9 | other |
| TMEM115 | 1.42E-05 | -2.426 | 2.90E-05 | -2.121 | transmembrane protein 115 | other |
| HOMER3 | 1.14E-03 | -2.424 | 3.77E-04 | -2.534 | homer homolog 3 (Drosophila) | other |
| HLA-C | 4.24E-05 | -2.398 | 4.35E-05 | -2.217 | major histocompatibility complex, class I, C | other |
| CD164 | 3.07E-04 | -2.377 | 7.49E-05 | -2.532 | CD164 molecule, sialomucin | other |
| FAT1 | 7.94E-03 | -2.373 | 3.71E-03 | -2.438 | FAT atypical cadherin 1 | other |
| DLGAP4 | 5.00E-04 | -2.366 | 3.76E-05 | -2.874 | discs, large (Drosophila) homolog-associated protein 4 | other |
| IFNGR2 | 6.94E-05 | -2.358 | 4.71E-06 | -2.812 | interferon gamma receptor 2 (interferon gamma transducer 1) | transmembrane receptor |
| TMEM50B | 4.07E-03 | -2.347 | 6.96E-04 | -2.707 | transmembrane protein 50B | other |
| GPR108 | 2.95E-04 | -2.345 | 4.53E-04 | -2.093 | G protein-coupled receptor 108 | G-protein coupled receptor |
| TM2D1 | 2.04E-04 | -2.314 | 1.06E-05 | -2.864 | TM2 domain containing 1 | G-protein coupled receptor |
| IL6ST | 3.31E-03 | -2.283 | 2.66E-03 | -2.177 | interleukin 6 signal transducer (gp130, oncostatin M receptor) | transmembrane receptor |
| ACTR3 | 2.94E-03 | -2.279 | 5.28E-04 | -2.58 | ARP3 actin-related protein 3 homolog (yeast) | other |
| FARP1 | 7.22E-05 | -2.257 | 1.07E-05 | -2.479 | FERM, RhoGEF (ARHGEF) and pleckstrin domain protein 1 (chondrocyte-derived) | other |
| PTGIR | 3.60E-03 | -2.228 | 2.59E-04 | -2.816 | prostaglandin I2 (prostacyclin) receptor (IP) | G-protein coupled receptor |
| SLC40A1 | 5.08E-04 | -2.221 | 3.25E-04 | -2.158 | solute carrier family 40 (iron-regulated transporter), member 1 | transporter |
| PDLIM4 | 1.40E-03 | -2.181 | 1.27E-03 | -2.058 | PDZ and LIM domain 4 | other |
| SNTA1 | 2.23E-04 | -2.172 | 2.36E-05 | -2.474 | syntrophin, alpha 1 | other |
| ICAM5 | 2.19E-03 | -2.159 | 8.85E-04 | -2.214 | intercellular adhesion molecule 5, telencephalin | other |
| ABCD1 | 7.11E-03 | -2.147 | 1.89E-03 | -2.348 | ATP-binding cassette, sub-family D (ALD), member 1 | transporter |
| BVES | 7.94E-07 | -2.126 | 1.39E-07 | -2.245 | blood vessel epicardial substance | other |
| TM9SF1 | 6.76E-03 | -2.11 | 5.86E-04 | -2.409 | transmembrane 9 superfamily member 1 | transporter |
| GRIA3 | 2.58E-03 | -2.105 | 1.30E-04 | -2.695 | glutamate receptor, ionotropic, AMPA 3 | ion channel |
| TENC1 | 2.19E-08 | -2.084 | 3.25E-09 | -2.21 | tensin like C1 domain containing phosphatase (tensin 2) | other |
| ADCY6 | 7.46E-03 | -2.05 | 2.24E-03 | -2.201 | adenylate cyclase 6 | enzyme |
