# Supplementary material for: CD44 Is a Negative Cell Surface Marker for Pluripotent Stem Cell Identification during Human Fibroblast Reprogramming
Source: PLoS One. 2014 Jan 9;9(1):e85419. doi: 10.1371/journal.pone.0085419 (PMC3887044; doi:10.1371/journal.pone.0085419)
Supplement: Table S2 — List of Wnt pathway genes that are differentially expressed in H9 ESCs or fully reprogrammed iPSCs compared to BJ fibroblasts. (DOCX) [file pone.0085419.s010.docx]

**Table S2:** List of Wnt pathway genes that are differentially expressed in H9 ESCs or fully reprogrammed iPSCs compared to BJ fibroblasts.

| **Symbol** | **H9 p-value** | **H9 fold change** | **FR p-value** | **FR fold change** | **Entrez Gene Name** | **Type(s)** |
| --- | --- | --- | --- | --- | --- | --- |
| ACVR1 | 7.65E-05 | -3.279 | 7.98E-05 | -2.942 | activin A receptor, type I | kinase |
| ACVR2B | 5.17E-06 | 3.189 | 1.16E-06 | 3.398 | activin A receptor, type IIB | kinase |
| AKT1 |  |  | 1.20E-05 | -2.213 | v-akt murine thymoma viral oncogene homolog 1 | kinase |
| APPL2 | 1.03E-07 | -5.086 | 2.51E-08 | -5.397 | adaptor protein, phosphotyrosine interaction, PH domain and leucine zipper containing 2 | other |
| AXIN2 | 3.25E-05 | 5.118 | 8.57E-06 | 5.524 | axin 2 | other |
| BCL9 | 1.61E-09 | 3.858 | 2.67E-09 | 3.253 | B-cell CLL/lymphoma 9 | other |
| BMPR2 | 1.40E-05 | -9.604 | 1.98E-05 | -7.332 | bone morphogenetic protein receptor, type II (serine/threonine kinase) | kinase |
| CD44 | 6.63E-06 | -46.705 | 2.37E-05 | -21.839 | CD44 molecule (Indian blood group) | enzyme |
| CDH1 | 8.94E-07 | 60.33 | 7.53E-08 | 110.007 | cadherin 1, type 1, E-cadherin (epithelial) | other |
| CDH3 | 1.52E-04 | 17.341 | 8.07E-06 | 35.008 | cadherin 3, type 1, P-cadherin (placental) | other |
| CSNK1D | 1.40E-04 | -2.704 | 1.93E-04 | -2.4 | casein kinase 1, delta | kinase |
| CSNK2A1 | 1.21E-06 | 2.437 | 2.03E-06 | 2.168 | casein kinase 2, alpha 1 polypeptide | kinase |
| DKK1 | 9.86E-05 | -32.508 | 1.23E-05 | -52.554 | dickkopf WNT signaling pathway inhibitor 1 | growth factor |
| DKK3 | 1.02E-07 | -16.834 | 3.12E-07 | -10.244 | dickkopf WNT signaling pathway inhibitor 3 | cytokine |
| DKKL1 | 3.66E-03 | 2.081 |  |  | dickkopf-like 1 | other |
| DVL3 | 1.73E-04 | -2.583 | 4.25E-05 | -2.751 | dishevelled segment polarity protein 3 | other |
| FRZB | 1.90E-03 | 7.953 | 1.99E-03 | 6.557 | frizzled-related protein | other |
| FZD2 |  |  | 6.69E-03 | -2.993 | frizzled family receptor 2 | G-protein coupled receptor |
| FZD3 | 6.53E-06 | 9.106 | 4.50E-06 | 8.102 | frizzled family receptor 3 | G-protein coupled receptor |
| FZD4 | 5.54E-04 | -3.374 | 8.85E-04 | -2.845 | frizzled family receptor 4 | G-protein coupled receptor |
| FZD5 | 1.10E-03 | 5.521 | 4.38E-04 | 5.774 | frizzled family receptor 5 | G-protein coupled receptor |
| FZD6 | 5.97E-05 | -2.872 | 7.80E-05 | -2.549 | frizzled family receptor 6 | G-protein coupled receptor |
| FZD8 | 3.83E-04 | 3.102 | 1.17E-03 | 2.453 | frizzled family receptor 8 | G-protein coupled receptor |
| FZD9 | 3.39E-05 | 3.343 | 1.41E-05 | 3.344 | frizzled family receptor 9 | G-protein coupled receptor |
| ILK | 1.73E-05 | -3.022 | 1.68E-05 | -2.753 | integrin-linked kinase | kinase |
| KREMEN2 | 1.91E-03 | 2.097 | 3.12E-04 | 2.348 | kringle containing transmembrane protein 2 | other |
| LRP1 | 2.54E-05 | -8.423 | 4.81E-05 | -6.162 | low density lipoprotein receptor-related protein 1 | transmembrane receptor |
| MAP4K1 | 8.32E-05 | 6.565 | 1.12E-04 | 5.266 | mitogen-activated protein kinase kinase kinase kinase 1 | kinase |
| NLK |  |  | 1.71E-04 | 2.35 | nemo-like kinase | kinase |
| NR5A2 |  |  | 5.35E-04 | 2.098 | nuclear receptor subfamily 5, group A, member 2 | ligand-dependent nuclear receptor |
| POU5F1 | 1.96E-07 | 25.252 | 6.04E-08 | 26.566 | POU class 5 homeobox 1 | transcription regulator |
| PPARD |  |  | 4.61E-09 | -2.032 | peroxisome proliferator-activated receptor delta | ligand-dependent nuclear receptor |
| PPM1J | 3.22E-05 | 2.86 | 1.55E-04 | 2.243 | protein phosphatase, Mg2+/Mn2+ dependent, 1J | phosphatase |
| PPP2R2B | 1.55E-06 | 7.654 | 7.71E-07 | 7.275 | protein phosphatase 2, regulatory subunit B, beta | phosphatase |
| PPP2R2C | 2.56E-04 | 2.47 | 5.75E-06 | 3.451 | protein phosphatase 2, regulatory subunit B, gamma | phosphatase |
| PPP2R3B | 7.92E-05 | 2.467 | 7.04E-05 | 2.305 | protein phosphatase 2, regulatory subunit B'', beta | phosphatase |
| PPP2R5B | 2.13E-05 | -2.614 | 6.68E-06 | -2.681 | protein phosphatase 2, regulatory subunit B', beta | phosphatase |
| RARA | 4.36E-05 | -2.44 | 9.96E-06 | -2.583 | retinoic acid receptor, alpha | ligand-dependent nuclear receptor |
| RARB |  |  | 8.75E-06 | -2.009 | retinoic acid receptor, beta | ligand-dependent nuclear receptor |
| SFRP2 | 3.48E-06 | 24.257 | 2.25E-06 | 20.848 | secreted frizzled-related protein 2 | transmembrane receptor |
| SMO |  |  | 2.14E-03 | 2.92 | smoothened, frizzled family receptor | G-protein coupled receptor |
| SOX2 | 5.10E-14 | 88.61 | 2.38E-14 | 78.572 | SRY (sex determining region Y)-box 2 | transcription regulator |
| SOX3 | 3.85E-07 | 27.5 | 2.18E-07 | 24.24 | SRY (sex determining region Y)-box 3 | transcription regulator |
| SOX4 | 1.39E-06 | 5.309 | 1.30E-06 | 4.636 | SRY (sex determining region Y)-box 4 | transcription regulator |
| SOX8 | 1.62E-03 | 4.489 | 9.11E-04 | 4.394 | SRY (sex determining region Y)-box 8 | transcription regulator |
| SOX9 | 4.26E-04 | 2.311 | 5.53E-04 | 2.097 | SRY (sex determining region Y)-box 9 | transcription regulator |
| SOX11 | 6.26E-08 | 11.786 | 1.66E-07 | 7.856 | SRY (sex determining region Y)-box 11 | transcription regulator |
| SOX13 | 5.23E-06 | 4.516 | 1.26E-06 | 4.851 | SRY (sex determining region Y)-box 13 | transcription regulator |
| SOX15 | 5.63E-03 | 5.36 | 1.98E-04 | 11.034 | SRY (sex determining region Y)-box 15 | transcription regulator |
| TCF3 | 3.96E-04 | 3.082 | 7.24E-04 | 2.594 | transcription factor 3 | transcription regulator |
| TCF7L1 | 5.36E-03 | 2.336 | 2.70E-03 | 2.363 | transcription factor 7-like 1 (T-cell specific, HMG-box) | transcription regulator |
| TGFB3 | 1.45E-05 | -4.005 | 2.31E-05 | -3.345 | transforming growth factor, beta 3 | growth factor |
| TGFBR2 | 8.66E-11 | -10.052 | 6.35E-11 | -8.7 | transforming growth factor, beta receptor II (70/80kDa) | kinase |
| TGFBR3 | 9.50E-05 | -3.987 | 1.07E-04 | -3.477 | transforming growth factor, beta receptor III | kinase |
| TLE1 | 1.20E-06 | 3.402 | 2.32E-07 | 3.681 | transducin-like enhancer of split 1 (E(sp1) homolog, Drosophila) | transcription regulator |
| TLE3 | 4.98E-04 | 2.325 |  |  | transducin-like enhancer of split 3 (E(sp1) homolog, Drosophila) | transcription regulator |
| TLE4 | 2.39E-04 | -2.784 | 2.54E-05 | -3.309 | transducin-like enhancer of split 4 (E(sp1) homolog, Drosophila) | transcription regulator |
| WNT2 | 3.99E-10 | -30.88 | 1.55E-10 | -29.868 | wingless-type MMTV integration site family member 2 | cytokine |
| WNT5A | 6.47E-10 | -108.347 | 2.18E-10 | -109.55 | wingless-type MMTV integration site family, member 5A | cytokine |
| WNT5B | 1.50E-08 | -12.529 | 1.12E-08 | -10.667 | wingless-type MMTV integration site family, member 5B | other |

*Blank spaces indicate that the gene had a fold change below the cut-off or was not significantly different from BJ fibroblasts.
