# Supplementary material for: CD44 Is a Negative Cell Surface Marker for Pluripotent Stem Cell Identification during Human Fibroblast Reprogramming
Source: PLoS One. 2014 Jan 9;9(1):e85419. doi: 10.1371/journal.pone.0085419 (PMC3887044; doi:10.1371/journal.pone.0085419)
Supplement: Table S3 — Selected genes that were differentially expressed in H9 ESCs and fully reprogrammed iPSCs compared to BJ fibroblasts. (DOCX) [file pone.0085419.s011.docx]

**Table S3:** Selected genes that were differentially expressed in H9 ESCs and fully reprogrammed iPSCs compared to BJ fibroblasts.

| **Symbol** | **H9 p-value** | **H9 fold change** | **FR p-value** | **FR fold change** |
| --- | --- | --- | --- | --- |
| NR2F2 | 0.00017548 | -14.028 | 1.54689E-05 | -22.969 |
| RGS4 | 3.28473E-07 | -8.690 | 9.23018E-07 | -6.003 |
| IL6ST | 0.00330701 | -2.283 | 0.0026594 | -2.177 |
| SNAI2 | 6.82245E-05 | -24.571 | 4.06515E-05 | -21.984 |
| NANOG | 1.67699E-05 | 16.919 | 4.63643E-06 | 18.868 |
